# Multifunctional Hybrid Gels Containing Silver Nanoparticles, Gold Nanorods, and Protoporphyrin IX for Synergistic Photothermal–Photodynamic Therapy

**DOI:** 10.1021/acsomega.6c02942

**Published:** 2026-08-06

**Authors:** Roberta C. S. Neves, Antônio L. de Oliveira, Silvany de S. Araújo, Palloma L. de Oliveira, Ana C. Brasileiro-Vidal, Iane B. V. Alves, José Y. R. Silva, Leonis L. Luz, Larissa T. Jesus, Ricardo O. Freire, Severino A. Junior

**Affiliations:** † Programa de pós-graduação em Ciências dos Materiais, 28116 Universidade Federal de Pernambuco UFPE, Recife, Pernambuco 50.740-560, Brazil; ‡ Departamento de Química, Universidade Estadual do Piauí, UESPI, Piripiri, Piauí 64.260-000, Brazil; § Departamento de Genética, UFPE, Recife, Pernambuco 50.670-901, Brazil; ∥ Departamento de Química Fundamental, UFPE, Recife, Pernambuco 50.740-560, Brazil; ⊥ Pople Computational Chemistry Laboratory, Department of Chemistry, UFS, São Cristóvão, Sergipe 49107-230, Brazil; # Núcleo Interdisciplinar de Ciências Exatas e da Natureza, CAA, Universidade Federal de Pernambuco, Nova Caruaru, Caruaru, Pernambuco 55.014-900, Brazil

## Abstract

Photodynamic and photothermal therapies, enhanced by nanotechnology, show great potential in skin cancer treatment. This study reports the synthesis and characterization of hybrid sodium alginate gels conjugated with protoporphyrin-IX, associated with gold and silver nanoparticles (AgNP/AuNR⊂PpIX-Alg). Silver nanoparticles improved singlet oxygen generation under a 660 nm light (quantum yield of 26.4%), while gold nanorods achieved a 54.3% photothermal conversion efficiency at 785 nm. Dual laser irradiation (660 and 785 nm) led to a strong synergistic therapeutic effect, reducing melanoma cell viability (B16–F10) to 9.83%, with no cytotoxicity to L929 fibroblast cells. These results support AgNP/AuNR⊂PpIX-Alg as a promising platform for multimodal cancer therapy.

## Graphical abstract

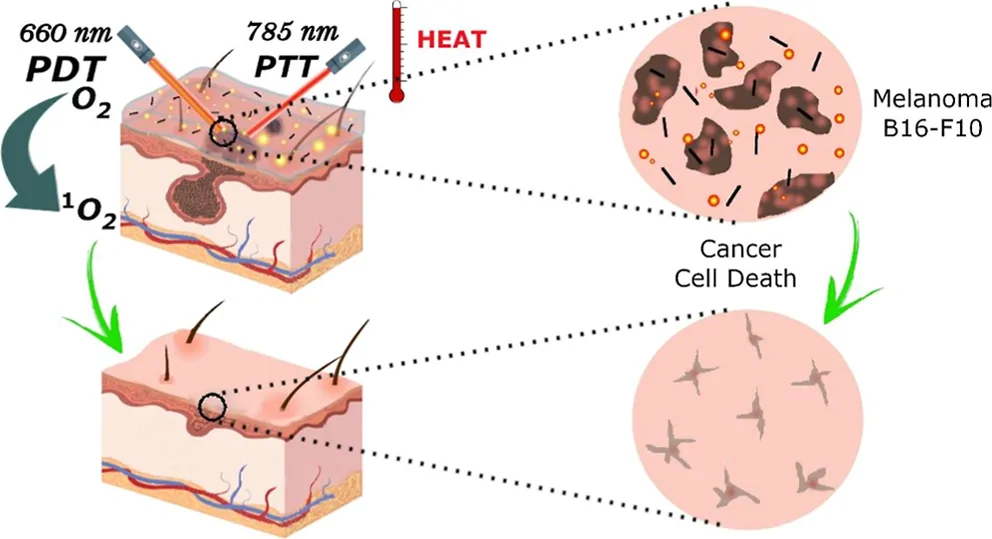

## 1 Introduction

The increasing incidence of cancer represents a major global public health and economic challenge. Cancer is a major cause of death worldwide, and the total global number of cases has been increasing.^1^ The American Cancer Society estimates that the incidence will reach 7180 people, with about 4600 men and 2580 women expected to die of melanoma.^2^ Skin cancer is the most common malignancy in the white population, with an estimated annual increase of around 3–7% over the past few decades.^3^

Photodynamic and photothermal therapies, combined with nanotechnology, are innovative technologies with broad perspectives for application in cancer treatment.^4–6^ Photodynamic therapy (PDT) as a first definitive treatment has attracted increasing attention, since it offers a minimally invasive and highly effective option for cancer treatment, due to the combination of photosensitizer, light, and local reactive oxygen species, as well as singlet oxygen (ROS) and radical generation. The use of near-infrared (NIR) light is particularly advantageous in this context, as the biological tissue presents reduced absorption and scattering in the so-called NIR biological transparency windows: the first window (NIR-I, ∼650–950 nm) and the second window (NIR-II, ∼1000–1700 nm),^7,8^ allowing for deeper light penetration into tissues with minimal photodamage to surrounding healthy cells. PDT therapy exploiting this spectral range has shown promising results.^4^

Protoporphyrin IX (PpIX) is a photosensitizing agent belonging to the porphyrin class and is clinically used in PDT because it presents cytotoxicity mediated by the formation of highly reactive oxygen species, such as singlet oxygen (^1^O_2_).^9^ Porphyrin compounds have been modified through functionalization to obtain well-conjugated hydrogels, which are promising carrier systems for the controlled diffusion of hydrophobic photosensitizers.^10^ Additionally, ablation via photothermal transduction has been employed based on local or even widespread temperature rise.

Jung and colleagues used nanographene oxide conjugated to hyaluronic acid as photothermal ablation therapy for melanoma skin cancer with a near-infrared (NIR) laser. The NIR irradiation completely ablated the tumor tissues without recurrence of tumorigenesis, which is promising for further research.^11^ Most importantly, the wavelength of the NIR light is in the “biological window”, which means that NIR light is minimally absorbed by the blood and soft tissues, allowing for deep penetration into the tissues.^12^ The use of near-infrared irradiation is particularly advantageous for PDT applications due to the greater tissue penetration achieved within the biological optical window. In the 660–785 nm spectral region, penetration depths in pig skin can reach up to 1.5 cm, increasing to approximately 3 cm between 1000 and 1220 nm, owing to reduced light scattering and lower absorption by endogenous chromophores.^13^ These methods are being widely applied in cancer treatment, which is motivating further research around the world.^14–16^

Currently, research on nanoparticles associated with polymeric hydrogels has increased due to their biocompatibility and exceptional physical and chemical properties, as well as their photodynamic action, which is difficult to obtain through a synthetic route.^17^ Au–Ag nanoparticles are commonly used in biomedical applications due to their excellent characteristics of water solubility, nontoxicity, noncarcinogenicity, and biodegradability,^5^ motivating the development of new technologies to target a variety of synergistic therapeutic strategies in health.^18^

A promising alternative for cancer treatment is the use of multifunctional platforms that combine materials with both photothermal therapy (PTT) and photodynamic therapy actions. Recent studies have shown that metal nanoparticles associated with polymeric hydrogels are efficient in NIR-mediated cell ablation, attenuating hypoxia status and synergistically reprogramming the tumor region.^19–21^ Within this perspective, alginates have been the subject of many studies. They are polysaccharides found in the intercellular matrix of brown algae, formed by units of mannuronic acid and guluronic acid, linked through bonds 1–4.^22^ Alginates can be used as hydrogels through three-dimensional cross-linked networks composed of hydrophilic polymers with high water content.^23,24^ Sodium alginate (SA) is a particularly interesting option due to its biodegradability, biocompatibility, low toxicity, low cost, and ability to easily associate with bivalent cations such as calcium (Ca^2+^), strontium (Sr^2+^), and barium (Ba^2+^). These cationic interactions are responsible for the formation of gels through safer junctions between alginate chains and divalent cations.^25^

Adding metallic components such as silver nanoparticles (AgNPs), gold nanospheres (AuNPs), and nanorods (AuNRs) to alginate-based hydrogels enhances their rheological properties and allows for chemical modifications. The functional groups in their structures increase the electrostatic, van der Waals, and hydrogen bonding between the polymer chains and nanoparticles.^26–28^ These features drive important biomedical applications, including drug therapies, regenerative bone tissue systems, and tissue engineering.^29–31^

Currently, covalently cross-linked alginate produces matrices with good mechanical properties, as well as stable and robust gel networks. Coupling agents, such as *N*-(3-dimethyl aminopropyl)-*N*′-ethyl carbodiimide hydrochloride (EDC) and *N*-hydroxysuccinimide (NHS), can be used in alginate synthesis by activating the carboxylic acid group, which favors cross-linking of gels. These can be used for a variety of biological applications such as drug delivery and photothermal and photodynamic therapies.^10,32–36^ Similar approaches are presented in the literature, combining fluorescence signals of biomaterials for diagnostic purposes, confirming the role of theranostic medicine.^37^

Many works have studied the interesting properties of metallic nanoparticles as photothermal agents,^38–41^ converting light into heat, leading to a rise in temperature and causing localized hyperthermia, selective “nanophotothermolysis”.^42^ Recently, branched-polydopamine-coated Au–Ag nanoparticles (Au–Ag@PDA) have emerged as a new therapeutic modality due to their high stability, high photothermal conversion efficiency, and in vitro and in vivo photothermal effects on cell lines, in particular, several cellular mechanisms, including dependent and independent caspase apoptosis, mitochondrial damage, lysosomal membrane permeability, and autophagy.^5^ More specifically, AuNRs are being extensively studied in PTT because they have excellent properties, such as the localized surface plasmon resonance (LSPR) and cover a wide spectral range, from the visible region to NIR.^42,43^

In the present work, we focus on these nanotechnological approaches, investigating, in vitro, two multifunctional hybrid gels based on alginate associated with AgNPs and AuNRs, conjugated to PpIX, against skin cancer, via PDT and PTT. We developed new multifunctional systems from cross-linked SA associated with AgNPs, AuNRs, and conjugated to the PpIX photosensitizer, obtained through a simplified and low-cost process. In the in vitro assays, with two cell lines, L929 mouse fibroblasts and melanoma B16–F10, we evaluated PDT, PTT, and chemo-photothermal effects. Multifunctional platforms with interesting synergistic effects are presented, simultaneously monitoring, demarcating, and treating murine melanoma cells (B16–F10) as a viable therapeutic strategy for skin cancer.

## 2 Experimental Section

### 2.1 Reactants

The reagents, ascorbic acid (99%), chloroauric acid (HAuCl_4_, 99,999%), sodium borohydride (NaBH_4_, 98%), sodium citrate (99%), cetyltrimethylammonium bromide (CTAB, 99%), polyvinylpyrrolidone (PVP) (MW = 100,000 g mol^–1^), *N*-(3-dimethyl aminopropyl)-*N*′-ethyl carbodiimide hydrochloride (EDC, >98%), *N*-hydroxysuccinimide (NHS, 98%), and methylene blue (MB, ≥82%), were acquired from Sigma-Aldrich (São Paulo, Brazil). Sulfuric acid (98%) was purchased from Micro-Chemistry (São Paulo, Brazil). Sodium alginate (NaAlg) of molar mass MW = 100,000 g·mol^–1^ and approximately 61% mannuronic acid and 39% guluronic acid, with an M/G ratio of 1.56. It was acquired from Sigma-Aldrich. All reactants were used without further purification.

### 2.2 Equipment and Measurement Conditions

The scanning electron microscopy (SEM) images were obtained in a TESCAN MIRA fourth generation with an FEG Schottky electron emission source, which combines SEM imaging and live elemental composition analysis. The analysis was performed by applying an acceleration voltage ranging from 15 to 25 kV, and the images were obtained using secondary electrons and backscattered secondary electrons. To perform SEM analysis, the gel samples were freeze-dried, and the powder samples were fixed to the sample holders (stubs) with carbon tape and then coated with a gold film using a sputtering coater. The Transmission Electron Microscopy (TEM) images were acquired in a JEM-2100 electron microscope from JEOL, operating at an acceleration voltage of 200 kV. Diluted aqueous solutions were carefully deposited on 400-mesh C-coated copper grids (Ted Pella Inc., USA) and dried in air. The ultraviolet–visible–near-infrared (UV–vis–NIR) diffuse reflectance spectra were obtained on a UV-2600-Shimadzu spectrophotometer (Recife, Brazil), with BaSO_4_ as the reflectance standard. The absorption spectra were obtained from diffuse reflectance using the Kubelka–Munk function *f*(*R*_∞_) = (1 – *R*_∞_)­2/2*R*_∞_ = *K*/*S*, where *f*(*R*_∞_) is referred to as the Kubelka–Munk function, *R*_∞_ is the absolute reflectance of the sample, *K* is the absorption coefficient, and *S* is the scattering coefficient. All samples were diluted in BaSO_4_ to perform data collection. Thermogravimetric analyses were performed in a DTG-60H, and the samples were filled under a flow of 50 mL/min of N_2_ with a temperature change rate of 10 °C/min (Shimadzu, from Recife, Brazil). FTIR spectra were acquired on a NEXUS-870 spectrophotometer (model Spectrum 400), in the frequency range from 4000 to 400 cm^–1^, through the FT-IR method with a UATR accessory (PerkinElmer from Recife, Brazil). ^1^H NMR spectra were measured in a VARIAN Unity Plus, operating at 300 MHz. The samples were solubilized in D_2_O (Alg gel material) or DMSO-*d*_6_ (all other materials) at room temperature (VARIAN Unity Plus, from Recife, Brazil). Rheological properties, at room temperature (25 °C), were analyzed on a rheometer-controlled stress DHR of TA Instruments, using the software Rheometer MCR 301. All measurements were performed using parallel plate geometry (50 mm diameter) at a gap of 100 μm. Oscillatory dynamics measurements were performed in the frequency scan mode, where the applied stress (τ) was constant at 0.4 or 5 Pa in the domain of the linear viscoelastic region, and the oscillation frequency (ω) (0.1 to 100 Hz) (Anton Paar from Recife, Brazil). As an excitation source in the experiment of SOG and PTT, a DMCABC Photon laser III and a Laser Multimodo I0785MB0600MS were used (Equipment DMC from Recife, Brazil).

### 2.3 Methods

#### 2.3.1 Synthesis of Gold Nanorods (AuNR)

*1st step* (seed growth)—Initially, 250 μL of HAuCl_4_ aqueous solution (10 mM) was added to 7.5 mL of CTAB solution (0.1 M) in a vial and kept under constant stirring for 5 min. Subsequently, 600 μL of NaBH_4_ aqueous solution (0.01 M) was added, and the mixture was kept under continuous stirring for 1 min.

*2nd step* (growth of the gold nanorods)—5 mL of HAuCl_4_ (10 mM), 2 mL of H_2_SO_4_ (0.5 M), 1 mL of AgNO_3_ (10 mM), and 0.8 mL of ascorbic acid (0.1 M) were added to 100 mL of CTAB, in this order, in a vial and kept under stirring until the mixture became colorless. Subsequently, 240 μL of seed solutions were added to this solution and kept at room temperature for 12 h. Finally, the AuNRs were collected by centrifugation and washed twice with distilled water.^44^ All solutions were prepared using ultrapure water.

#### 2.3.2 Synthesis of Silver Nanoparticles

125 mL of the aqueous solution of AgNO_3_ (1 mM) was heated under reflux and stirred until boiling. Then, 300 μL of a 1% w/v polyvinylpyrrolidone aqueous solution was added to this reflux system. After mixing, 5 mL (rate of 1 mL/min) of sodium citrate (1% w/v) was added to the mixture while continuously boiling until it took on a pale-yellow color, indicating the formation of AgNPs. This solution was cooled under stirring in a thermostatic bath at 22 °C.^45,46^ Finally, the AgNPs were collected by centrifugation and washed twice with distilled water.

#### 2.3.3 Estimation of the Metal Concentration in the Metal-Nanoparticle Suspensions

To estimate the silver and gold concentrations in the synthesized suspensions of the AgNPs and ANRs, we have used standardized photometric methods based on extinction coefficients and absorbance at the surface plasmon absorption. For the silver quantification, we have used the extinction coefficient of 3.36 × 10^10^ L mol^–1^ cm^–1^ and the absorbance at 412 nm (*A* = 0.5).^76^ For gold quantification, we have used an aspect ratio of 4.4, which leads to an extinction coefficient of 4.6 × 10^9^ L mol^–1^ cm^–1^, and absorbance of longitudinal plasmon at 910 nm (*A* = 1.35).^77^ The concentrations of Ag and Au in the colloidal and hybrid gels are listed in Table S2. More detail on silver and gold quantification is presented in the Supporting Information.

#### 2.3.4 Preparation of the Alginate Hydrogel (Alg)

The alginate hydrogel was obtained according to the approach outlined by Rassu et al.^47^ Following this method, we dissolved 0.4 g of NaAlg in 10 mL of ultrapure water under vigorous stirring for 3 h at 40 °C.

#### 2.3.5 Preparation of the Alg-Conjugated Protoporphyrin Gel (PpIX-Alg)

Initially, 12 mg of PpIX was added to 4.2 mL of DMSO. Then, 6 mg of EDC and 3 mg of NHS were introduced for activation of PpIX carboxylic groups.^48^ Next, 4 mL of ethylenediamine (EDA) was added, and stirring was maintained for 24 h at 60 °C.^49^ The resulting brown precipitate (PpIX-EDA) was separated by centrifugation, washed with ultrapure water three times, and dispersed in water to a 1 mL suspension (suspension A). Afterward, 0.2 g of NaAlg, 0.1 g of EDC, and 0.03 g of NHS were mixed in 4 mL of highly pure water for 3 h under constant stirring at 40 °C, in a closed vial.^49^ Then, suspension A was added, and stirring and heating were maintained for 6 h.

#### 2.3.6 Synthesis of AgNP⊂PpIX-Alg and AgNP/AuNR⊂PpIX-Alg Hybrids

To obtain the metal-nanoparticle⊂PpIX-Alg (MNP⊂PpIX-Alg) hybrid gels, we used the same procedure as for PpIX-Alg, using metal-nanoparticle suspensions as the solvent for NaAlg. Thus, after the coupling of PpIX to EDA step, PpIX-EDA was dispersed in water to 1 mL of the suspension (suspension A). Afterward, 0.2 g of NaAlg, 0.1 g of EDC, and 0.03 g of NHS were mixed in the corresponding metal nanoparticle suspension for 3 h under constant stirring at 40 °C, in a closed vial. Then, suspension A was added, and stirring and heating were maintained for another 6 h. The volume of metal nanoparticle suspensions is shown in Table S1.

#### 2.3.7 Singlet Oxygen (^1^O_2_) Generation

Twelve milligrams of the AgNP/AuNR⊂PpIX-Alg hybrid gel were diluted in 2 mL of H_2_O/DMSO (800 μL/1200 μL). Then, 2 mL of a solution of 1,3-diphenylisobenzofuran (DPBF, 0.7 mM in DMSO) was added. This was irradiated with a continuous-wave (CW) diode laser (λ_ex_ = 660 nm) at a power of 30 mW at intervals of 5 to 35 min (9.2–64.4 J cm^2^). DPBF was employed for the detection of singlet oxygen (probe), in which the absorbance spectrum was acquired every five seconds of irradiation. The quantum yields (Φ_s_) of singlet oxygen generation were determined by monitoring the oxidation of the probe DPBF at an absorbance of 414 nm.^50^

#### 2.3.8 Photothermal Conversion Experiments

The photothermal conversion measurements were performed by monitoring the temperature of the gels under irradiation with a NIR CW laser (λ = 785 nm) with power ranging from 0.5 to 1 W. The heating curve was obtained with the laser ON for 5 min, and the cooling curve with the laser OFF until it reached room temperature. The temperature was monitored with a thermal imaging camera FLIR E4 at intervals of 5 s on the heating ramp and 10 s on the cooling ramp. To estimate the photothermal conversion efficiency (η), we used eq 1, according to Roper’s approach^51,52^

1 $$\eta =\frac{hS\left(T_{\max}-T_{vis}\right)-Q_{0}}{I\left(1-1.10^{-A_{785}}\right)}$$

where *h* is the heat-transfer coefficient, *S* is the cross section of irradiated area, *T*_max_ is the maximum temperature at equilibrium of the hybrid gel, *T*_vis_ is the ambient temperature, *Q*_0_ is associated with the heat generated only by the full quartz cuvette of water under irradiation with the laser (1 W), *A*_785_ is the absorbance of the hybrid gel (*A*_785_ = 0.55), and *I* is the incident laser power (1 W). The value of *hS* was calculated from eq 2.^52^

2 $$\tau_{S}=\frac{\sum_{i}{m_{i}C}_{p,i}}{hS}$$

where τ_s_ is the system time constant represented by the slope of the cooling curve as the plot τ_s_ × ln θ; *m*_*i*_ and *C*_*p*,i_ are the mass and heat capacity of the water (80 mg and 418 J g^–1^ K^–1^) and quartz cell (1.62 g and 0.89 J g^–1^ K^–1^), respectively.

#### 2.3.9 In Vitro Assay

##### 2.3.9.1 Cell Lines and Culture Conditions

In this study, we used two cell lines, immortalized mouse fibroblasts (L929) and a murine melanoma cancer cell line (B16–F10), in order to verify whether conjugated hybrid gels of PpIX-Alg, AgNP⊂PpIX-Alg, and AgNP/AuNR⊂PpIX-Alg showed selectivity for cancer cells. L929 cells were cultured in DMEM and B16–F10 cells in RPMI 1640 media, which contained 10% fetal bovine serum (FBS), and they were incubated under a humidified atmosphere for 24 h (37 °C, 5% CO_2_).

##### 2.3.9.2 Effect of Lasers on Cell Viability

The effects of 660 and 785 nm CW lasers, used for singlet oxygen (^1^O_2_) generation and the photothermal conversion assay, under a power density ranging from 800 to 1000 mW, were examined for L929 and B16–F10 cells using an MTT assay, according to the methodology described by Silva et al.^53^ The cells were seeded into 96-well plates (2 × 10^4^ cells per well) and irradiated for 5 min with a 660 nm CW laser (30 mW) only, then a 785 nm CW laser only under different power densities, and finally, simultaneously, under the same conditions. After 24 h, all of the supernatants were removed, and the cells in the 96-well plate were further treated with 20 μL of MTT (0.5 mg mL^–1^) and 180 μL of culture media for 3 h. Subsequently, the MTT medium was carefully removed and 100 μL of DMSO was added into each well to read absorbance at 578 nm on a multidetection microplate reader. Results were expressed as the percentage of viable cells compared with untreated control cells (100%). All these tests were performed in triplicate.

##### 2.3.9.3 In Vitro Cytotoxicity

The cytotoxicity of PpIX, PpIX-Alg, AgNP⊂PpIX-Alg, and AgNP/AuNR⊂PpIX-Alg was evaluated on L929 and B16–F10 cells using an MTT assay. Cells were cultured under the conditions already mentioned. The cells were treated with PpIX, PpIX-Alg, AgNP⊂PpIX-Alg, and AgNP/AuNR⊂PpIX-Alg diluted to the concentrations of 2.06, 4.12, 8.2, 16.5, and 33 mg mL^–1^ of the gels for 48 h. Then, the MTT assay was performed as described above.

##### 2.3.9.4 Effect of Photodynamic, Chemo-, and Photothermal Therapy

To evaluate the combined effect of the photodynamic, chemo-, and photothermal therapy in the in vitro assay, the AgNP⊂PpIX-Alg and AgNP/AuNR⊂PpIX-Alg materials were selected and diluted to concentrations of 8.25 and 16.5 mg mL^–1^ of the gels, respectively. L929 and B16–F10 cells were seeded in 96-well plates at a concentration of 2 × 10^4^ cells/well and then treated with the hybrids described above for 24 h. Subsequently, the experiment was divided into four groups: control (cells + culture medium); AgNP⊂PpIX-Alg and AgNP/AuNR⊂PpIX-Alg irradiated with 660 nm CWL (30 mW cm^–2^); AgNP⊂PpIX-Alg and AgNP/AuNR⊂PpIX-Alg irradiated with 785 nm CWL (1 W cm^–2^); and AgNP⊂PpIX-Alg and AgNP/AuNR⊂PpIX-Alg simultaneously irradiated with 660 CWL and 785 nm CWL. The irradiation time for all experiments was 5 min. After this, the cells were incubated again for more than 24 h; then, 20 μL of MTT (0.5 mg mL^–1^) was added to the wells; and they were incubated for more than 3 h to measure cell viability.

#### 2.3.10 Statistical Analysis

Cytotoxicity was expressed as the percentage of viable cells against that of the untreated control cells. The data were expressed as mean ± standard deviation (SD). The normality and homogeneity of cell viability data were analyzed by the Shapiro–Wilk and Levene tests, respectively. After photodynamic and photothermal and photodynamic and chemo-photothermal therapies, cell viability data showed normal distribution, being compared statistically using parametric Tukey’s test. Meanwhile, the Mann–Whitney test (nonparametric) was used for cytotoxicity data of PpIX, PpIX-Alg, AgNP⊂PpIX-Alg, and AgNP/AuNR⊂PpIX-Alg. Statistical analyses were performed using the software STATISTICA 8.0 (*p* < 0.05), Statistica for Windows (Data Analysis Software System), Version 8.0. Computer Program Manual, Quick Reference. Statsoft. Inc., Tulsa.^54^

### 2.4 Theoretical Calculations

To obtain the macromolecule used for drug anchoring, a network composed of alginate and porphyrin molecules (ALGp), an initial anchoring process was carried out between alginate and porphyrin, following the adopted experimental methodology. In this first step, molecular docking was performed using alginate as the macromolecule and porphyrin as the ligand. Subsequently, the lowest-energy conformation of the alginate–porphyrin complex obtained in this step was used as the new macromolecular structure for additional docking calculations, aiming to identify other potential interaction sites between the porphyrin and alginate. All docking simulations were conducted using the AutoDock 4.2 software.^55^

The systems were prepared in AutoGrid4 ^55^ during a preliminary docking stage, where the size and position of the interaction grid box were defined. This box was used to map the interaction energies between different atom types present in the ligand and macromolecule.

The simulation for obtaining the ALGp system was conducted through a four-step procedure, in which one porphyrin molecule was added at each stage. For all steps, a grid box with average dimensions of 40 × 40 × 58 Å was defined to ensure adequate coverage of the interaction region.

It is important to note that, during the docking procedure, the interaction energy maps generated in the preliminary docking stage were used to calculate the total interaction energy between the evaluated systems. A rigid macromolecule–flexible ligand docking approach was employed, where the structure selected as the macromolecule was kept entirely rigid throughout the simulation, while the ligand (porphyrin) was modeled as flexible and oriented toward the macromolecular surface.

The docking simulations were performed using the Lamarckian Genetic Algorithm (LGA), with the following parameters: an initial population of 100 randomly generated individuals, a maximum of 27,000 generations, mutation and crossover rates of 0.02 and 0.08, respectively, and a maximum of 2.5 × 10^6^ energy evaluations.

Conformational clustering was performed based on root-mean-square deviation (RMSD), with conformations grouped and ranked in the ascending order of binding energy. Structures exhibiting an RMSD of less than 2 Å upon superposition were considered part of the same cluster.

Following the construction of the two-dimensional ALGp network, it was employed as a macromolecular structure to investigate potential interaction sites with chemotherapeutic agents commonly used in cancer treatment. The selected drugs for this analysis were doxorubicin, fluorouracil, and methotrexate.

The docking simulations for each drug were conducted by using the same methodological approach adopted during the assembly of the two-dimensional network, including identical algorithmic parameters and conformational clustering criteria.

In all cases, the defined grid box size was adequate to encompass the predicted interaction region between the macromolecule and the ligand. For consistency across all docking studies involving the three drugs, a uniform grid box dimension of 40 × 40 × 40 Å was utilized.

## 3 Results and Discussion

### 3.1 FTIR Spectroscopy and ^1^H NMR

The reaction mechanism of coupling PpIX to the Alg chain involves the prefunctionalization of the PpIX with ethylenediamine to create free amine sites (step 1), which will subsequently react with Alg (step 2), as shown in Figure 1.

**Figure 1:**
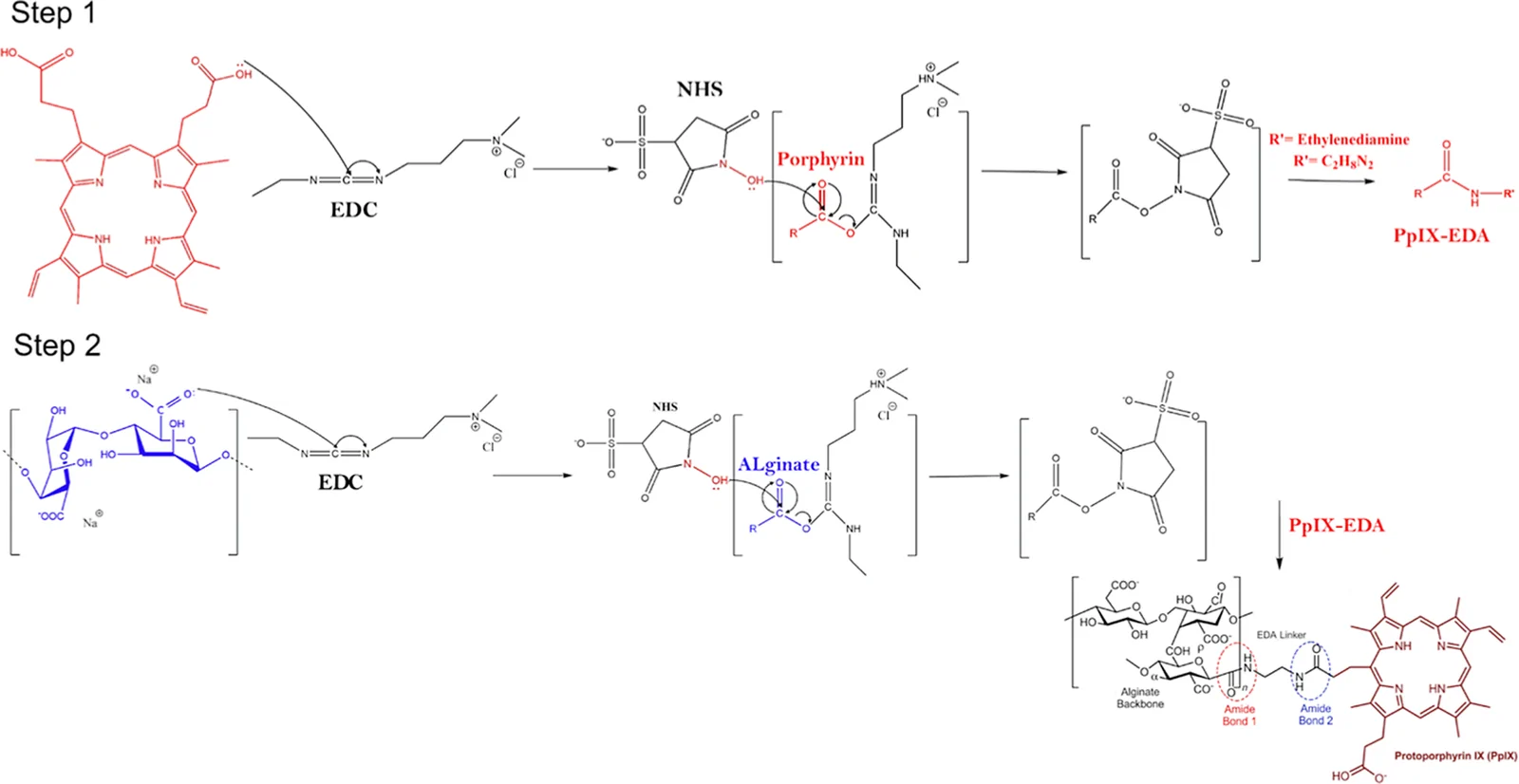
Schematic representation of the reaction mechanism involved in the conjugation of the protoporphyrin IX to the alginate chain.

In the first step, PpIX is dissolved in DMSO to ensure the carboxyl groups are accessible and reactive. After addition of EDC and NHS, EDC activates the two carboxyl groups of the porphyrin, and the NHS transforms them into active NHS esters. Ethylenediamine (EDA) is added in a large molar excess to ensure that only one end of the EDA molecule reacts with the porphyrin, leaving the other end free, forming PpIX-EDA, and preventing a single EDA molecule from bridging two porphyrin molecules. In step 2, EDC/NHS activates carboxyl groups along the polymer chain of Alg. Finally, PpIX-EDA (from Step 1) is added to the activated alginate solution. The free amine groups at the end of the porphyrin’s spacer attack the NHS esters on the alginate. The final amide bond is formed, resulting in the alginate-EDA-PpIX complex (PpIX-Alg).

To monitor the success of the coupling reaction steps, FTIR analyses were conducted on the EDC + NHS solution, PpIX, AgNP/AuNR⊂PpIX-Alg, AgNP⊂PpIX-Alg, PpIX-Alg, and Alg gels (Figure 2). The spectrum of the lyophilized Alg gel displays a broad band ranging from 3000 to 3700 cm^–1^, which is attributed to the O–H stretching vibrations of the water molecules. Two small bands can be observed at 2925 and 2850 cm^–1^, which are related to the C–H stretching vibrations of the saccharide structure and –CH_2_–, respectively. Other characteristic peaks at 1595 and 1410 cm^–1^ are assigned to asymmetric and symmetric stretching vibrations of the carboxylate (COO−) group, respectively.^56,57^ Also, signals of symmetric and asymmetric vibrations of the C–O–C bonds typical of alginate rings are exhibited at 1025 and 1085 cm^–1^, respectively. On the other hand, the spectrum of PpIX exhibits characteristic bands of tetraphenylporphyrin at 3315 cm^–1^, related to N–H axial deformation of the amine, and in the range of 2750–3250 cm^–1^, signals related to O–H and C–H aromatic stretching vibrations are verified. The intense band at 1695 cm^–1^, assigned to the stretching vibration of C=O, and the band at 1417 cm^–1^, assigned to C–O–H, result from normal vibration modes of carboxylic acid groups of PpIX.^58–60^

**Figure 2:**
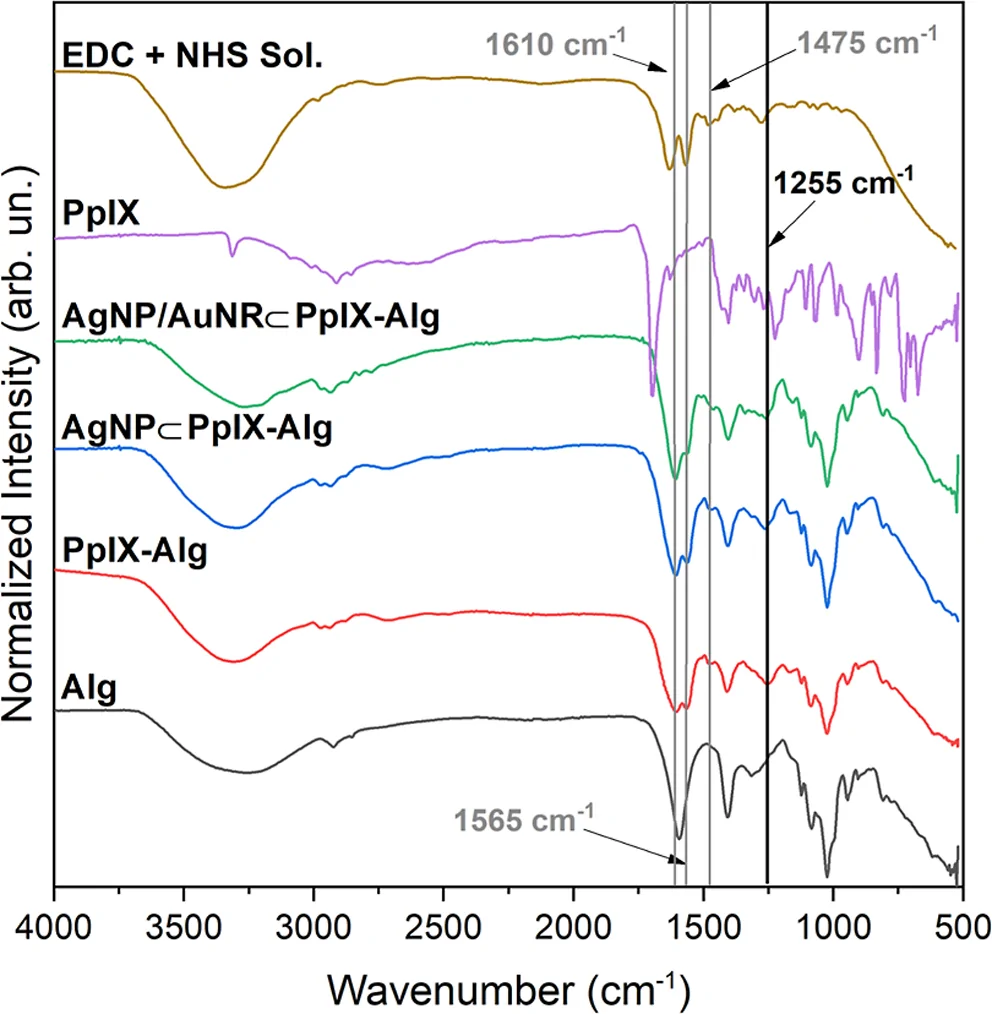
Fourier-transform infrared (FTIR) spectra of EDC + NHS sol, PpIX, hybrid gel of AgNP/AuNR⊂PpIX-Alg, AgNP⊂PpIX-Alg, PpIX-Alg, and Alg.

After conjugation, both the conjugated PpIX-Alg gel and AgNP⊂PpIX-Alg and AgNP/AuNR⊂PpIX-Alg hybrid gels displayed additional bands at 1610 and 1565 cm^–1^, compared to individual PpIX and Alg, where the bands related to pristine carboxylic acids are absent. These two bands are a strong indication that successful of formation of amide bonds between spacer EDA and PpIX and Alg and can be assigned to the carbonyl stretching.^61^ The coupling agents EDC/NHS also display the vibrational stretching at the frequencies of 1635 and 1570 cm^–1^; however, the signals in the hybrids showed bands with lower resolution and broadening, suggesting the superimposition of many frequency modes, i.e., those resulting from amide carbonyl stretching and possible agent coupling EDC/NHS. Another new band, at 1255 cm^–1^, can be assigned to C–N relative to amide and N–H bending. This reinforces the successful conjugation between the Alg chain and PpIX molecule through formation of amide bonds with ethylenediamine.

The conjugation between alginate chains and PpIX molecules was further confirmed by ^1^H NMR analysis (Figure 3). The ^1^H NMR spectra of alginate (indicated by the black line in Figure 3) exhibit signals corresponding to the β-d-mannuronate (M) and α-l-guluronate (G) chains, with M and G being isomers.^62^ The signal at 5.08 ppm is attributed to the anomeric hydrogen of the guluronic units. Peaks in the vicinity of 4.95 ppm are assigned to the ring proton of alginate.^62^ The intense signal around 4.6–4.9 ppm is attributed to HOD. For PpIX (red line in Figure 3), in the downfield region, the meso protons of the porphyrin ring (position x at the structure in Figure S1) were identified between δ 9.6 and 10 ppm. The internal vinyl protons (−CH=; position 7 at the structure in Figure S1) appeared between δ 6.1 and 8.35 ppm as multiplets, whereas the terminal vinyl protons (=CH_2_; positions 8a and 8b at the structure in Figure S1) were observed between δ 6.0 and 6.4 ppm.^63^ Additionally, the broad and low-intensity peak observed around 11.5–13 ppm is assigned to the proton of the carboxyl groups (position 9 at the structure in Figure S1). The proton signal of carboxylic groups is typically weak, broad, and often undetectable due to fluxionality (fast chemical exchange) and strong hydrogen bonding, as widely reported for carboxylic acid groups in solution.^64,65^ The peripheral methyl groups directly attached to the pyrrolic units (position 6 at the structure in Figure S1) produced resonances in the δ 3.3–3.55 ppm region. The propionic side chains exhibited characteristic aliphatic methylene resonances in two distinct regions. The α-methylene protons (position 7 at the structure in Figure S1) adjacent to the porphyrinic ring appeared between δ 4.15 and 4.3 ppm, due to the influence of the neighboring aromatic π-system. In contrast, the β-methylene protons (position 8 at the structure in Figure S1) adjacent to the carboxylic groups resonated between δ 3.0 and 3.2 ppm.

**Figure 3:**
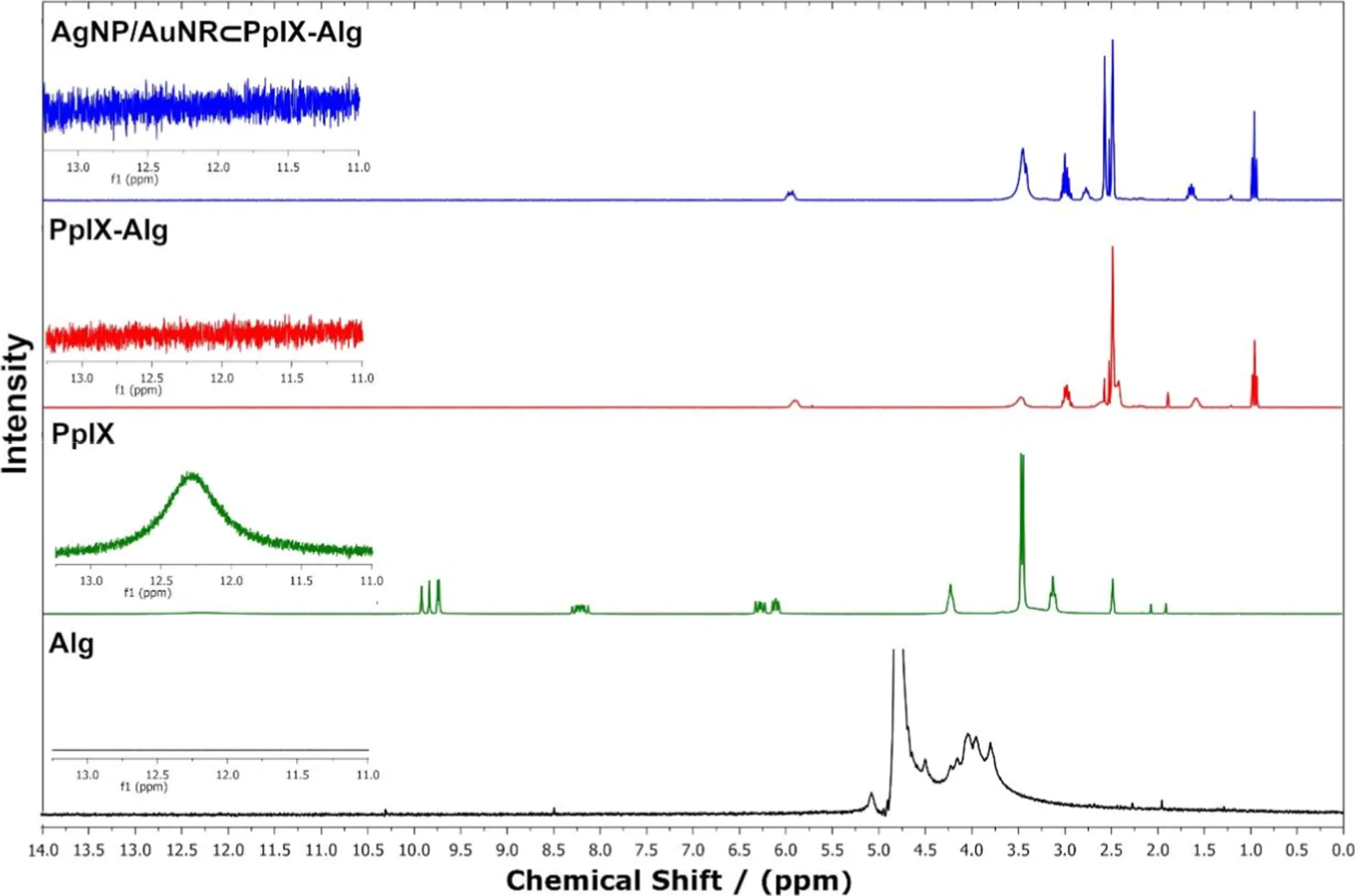
^1^H NMR spectra of alginate, PpIX (DMSO-*d*_6_), PpIX-Alg (DMSO-*d*_6_), and AgNP/AuNR⊂PpIX-Alg (DMSO-*d*_6_).

Conversely, in the ^1^H NMR spectra of the conjugated PpIX-Alg and hybrid gels, the peaks corresponding to the carboxyl protons of PpIX (as shown in Figure 3) are absent, suggesting that a reaction has taken place at the carboxyl sites. This is due to the conjugation between the alginate chain and PpIX, made through amide-bond formation with the EDA spacer. Some peaks in the range of 3.1–0.9 ppm of the hybrid are assigned to EDC and NHS (see Figures S2 and S3). The broadening of certain peaks is attributed to the formation of hydrogen bonds and an increase in viscosity.^66^ A low-intensity peak in the range of 2.72–2.84 ppm is assigned to the methylene group of the ethylene diamine used as a binder in covalent conjugation (see Figure S4).^66–68^ This signal is more pronounced in the AgNP/AuNR⊂PpIX-Alg spectrum. It is important to note that the occurrence of proton signals of the methylene group of EDA is only possible if PpIX is conjugated to that after the first coupling step procedure.

#### 3.1.1 Molecular Docking—Study of the Interactions between Drugs and Chain of Sodium Alginate Conjugated with Protoporphyrin-IX

The experimental results demonstrated the efficacy of the hybrid gels conjugated with porphyrin and alginate for the treatment of skin cancer. Consequently, it is hypothesized that incorporating drugs already established in this therapeutic area could further enhance the potential and effectiveness of the developed systems. To investigate this, the binding mode and interaction energy between the multifunctional system and the three anticancer drugs were evaluated.

Considering that the drugs were treated with flexible torsional and dihedral angles, multiple conformations were sampled during the conformational search and energy calculations. A total of 100 conformations were analyzed, ranked according to ascending interaction energy, and clustered based on the root-mean-square deviation (RMSD). The clustering results are presented in a bar chart as depicted in Figure 4.

**Figure 4:**
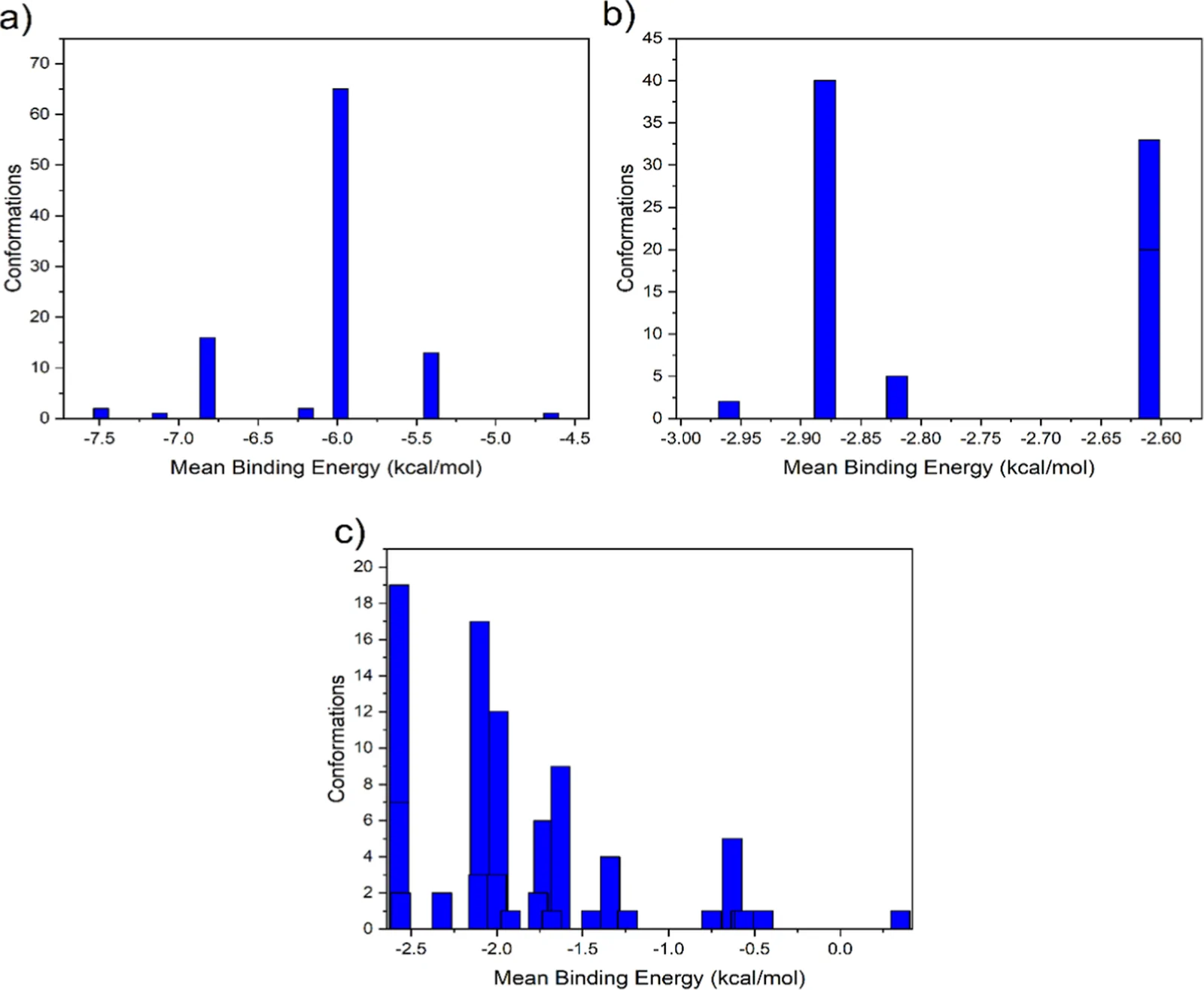
Bar charts depicting the clustering of distinct simulated conformations corresponding to the interactions between the drugs and the ALGp network: (a) doxorubicin, (b) fluorouracil, and (c) methotrexate.

An analysis of Figure 4a reveals that 65% of the structures exhibited similar conformations, characterized by a root-mean-square deviation (RMSD) below 2 Å and an average binding energy of −5.98 kcal/mol. Notably, the conformation with the lowest energy within this cluster presented an interaction energy of −7.14 kcal/mol. Smaller clusters containing fewer conformations were also identified, some with even lower average binding energies; however, the nature of the interactions observed remained consistent across clusters. Given the high proportion of conformations clustered in specific regions alongside the low calculated interaction energies, these clusters are likely to represent the most probable binding modes between doxorubicin and the ALGp network.

A similar analysis was conducted for the results obtained from the interaction calculations between the ALGp network and the compounds fluorouracil and methotrexate. In the case of Figure 4b, the largest cluster, comprising 40% of the structures, exhibited an average binding energy of −2.88 kcal/mol, with the most stable conformation reaching a binding energy of −2.98 kcal/mol.

For Figure 4c, which presents the interaction results with methotrexate, a considerably higher number of clusters were observed. This outcome is likely attributable to the molecular structure of methotrexate, which contains branches with single bonds, conferring greater rotational freedom to the molecule and, consequently, increasing the mean overlap error during clustering. Despite this, the interaction patterns remained consistent across the clusters, with lower percentages of structures.

In this scenario, the largest cluster accounted for 19% of the structures, with an average binding energy of −2.57 kcal/mol, while the lowest-energy conformation reached −3.58 kcal/mol. Notably, the cluster with the lowest percentage of structures coincided with the one presenting the most favorable binding-energy value.

In molecular docking, the average binding energy represents the strength and stability of the interaction between a ligand molecule and a target molecule. This value reflects how strongly the ligand is attracted to or retained by the target at its binding site, allowing for the prediction of the ligand’s affinity and potential efficacy. More negative energy values indicate a stronger and more stable interaction between the ligand and the target. This energy takes into account van der Waals forces, electrostatic interactions, and hydrogen bonding, among other factors.^69,70^ In this context, the three evaluated drugs demonstrated favorable interactions with the ALGp system. When comparing the docking results of the evaluated drugs with the developed system, it was observed that doxorubicin exhibited the lowest binding energy, and consequently, the most favorable interaction with ALGp.

Figure 5 presents the predicted interaction modes between the evaluated systems obtained through molecular docking calculations. Additionally, this figure displays the overlap of the conformations grouped within the most populated cluster for each drug: 65 conformations for doxorubicin, 40 for fluorouracil, and 19 for methotrexate. It is important to highlight that the conformation with the lowest binding energy is colored cyan.

**Figure 5:**
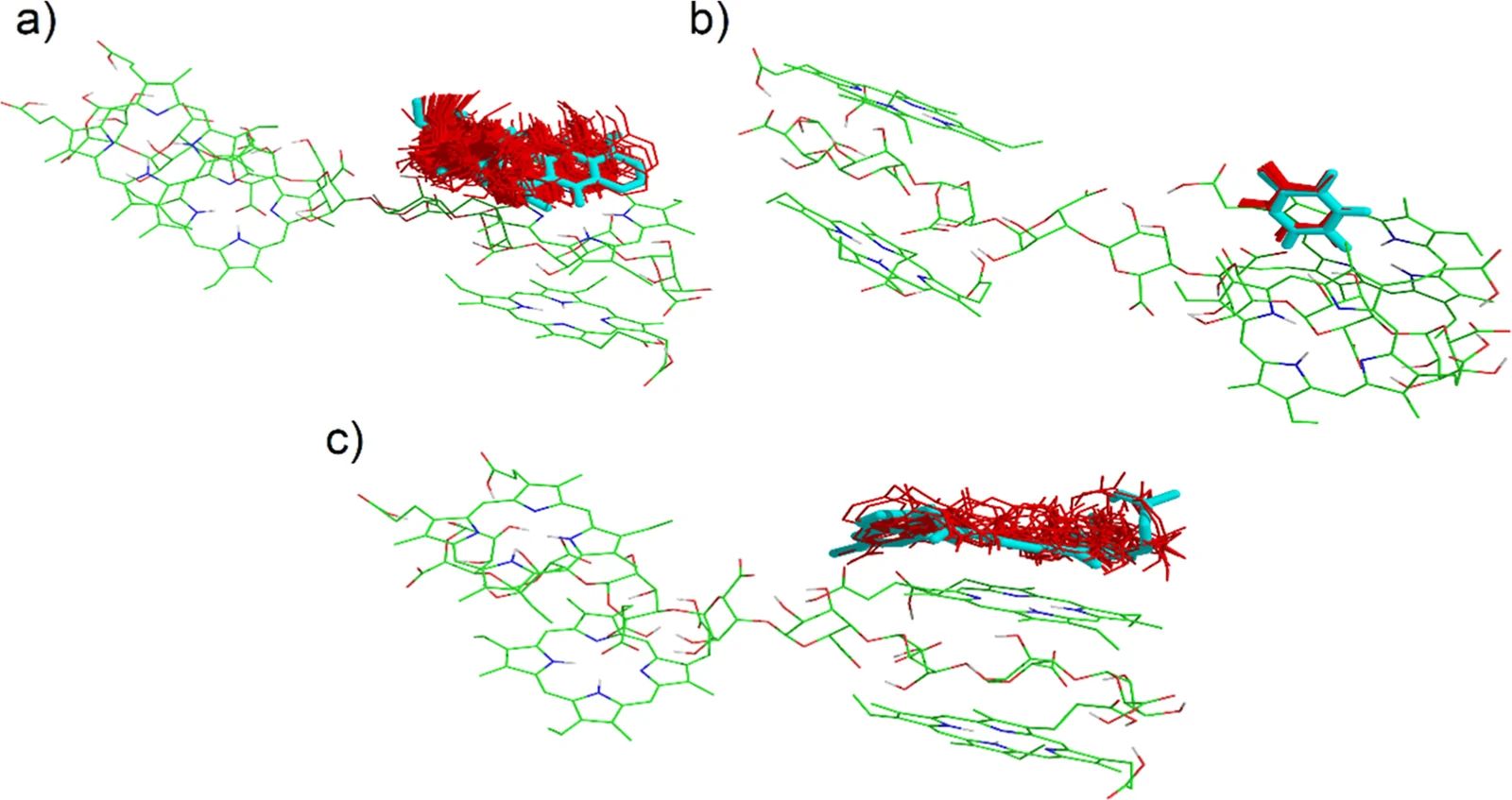
Overlay of the clustered conformations from the docking calculation: (a) doxorubicin, (b) fluorouracil, and (c) methotrexate.

As the ALGp network was modeled in a two-dimensional configuration, no pores were formed, and the interaction analysis was performed by simulating a surface area. In this context, several potential interaction sites can be considered when structurally evaluating alginate and porphyrin, including hydroxyl group interactions, hydrogen bonding, and π–π stacking interactions. Based on the lowest-energy conformation, the specific interactions and their corresponding distances were identified and analyzed, as illustrated in Figure 6.

**Figure 6:**
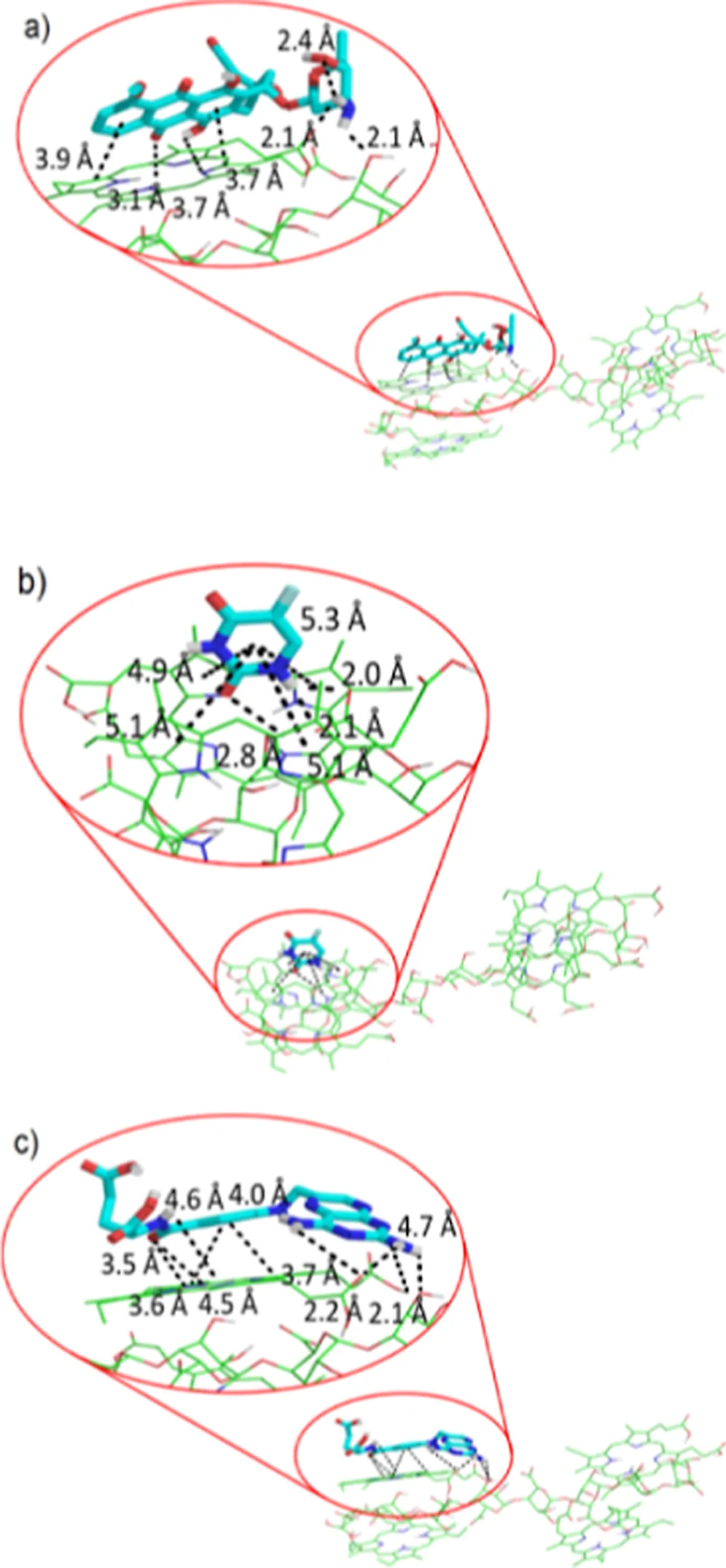
Interaction distances between the lowest-energy conformation of the drugs and the ALGp network, obtained from the docking simulation of the cluster with the highest number of conformations: (a) doxorubicin, (b) fluorouracil, and (c) methotrexate.

Initially, it is important to emphasize that all three drugs exhibited interactions with both the porphyrin and alginate molecules. The main types of interactions identified were hydrogen bonding and π–π stacking. Notably, π–π stacking interactions are commonly observed in adsorption processes. The results indicate the presence of such interactions involving aromatic rings in both molecular structures, with estimated intermolecular distances ranging from 3.9 to 5.1 Å. This distance range is consistent with the findings reported by Headen et al., whose study on π–π stacking interactions concluded that the interaction between ring centers predominantly occurs at distances between 4.0 and 8.0 Å.^104^

Furthermore, it was observed that the interaction distances between the doxorubicin molecule and the ALGp network were shorter compared with the other compounds, which may be associated with its lower binding energy.

As reported in the literature, these drugs demonstrate high efficacy and are widely recognized for their effectiveness in the treatment of cancer. The multifunctional network synthesized, thoroughly characterized, and evaluated in this study exhibited a remarkable chemotherapeutic activity. Consequently, it is reasonable to assume that the adsorption of these drugs onto this network may further enhance their therapeutic efficacy in the treatment of cancer.

### 3.2 Rheological Behavior

Figure 7 displays the storage (*G*′) and loss (*G*″) moduli from the frequency sweep experiment performed for the Alg, conjugated Alg, hybrids, and conjugated hybrid gels. Verification of the Alg and hybrid gels as the *G*″ > G′ in the angular frequency range from 0.1 up to 130 rad·s^–1^ (Figure S6a). This suggests that the materials behave as a viscoelastic liquid in this region. After 130 rad·s^–1^ (*G*′ = *G*″), *G*′ becomes greater than *G*″, behaving as a viscoelastic solid material.^72^ The rotational experiment indicated that the viscosity of the Alg and hybrid gels was reduced at an increasing shear rate in the range of 0–200 s^–1^, as common to a typical non-Newtonian fluid (Figure S6b).^73,74^

**Figure 7:**
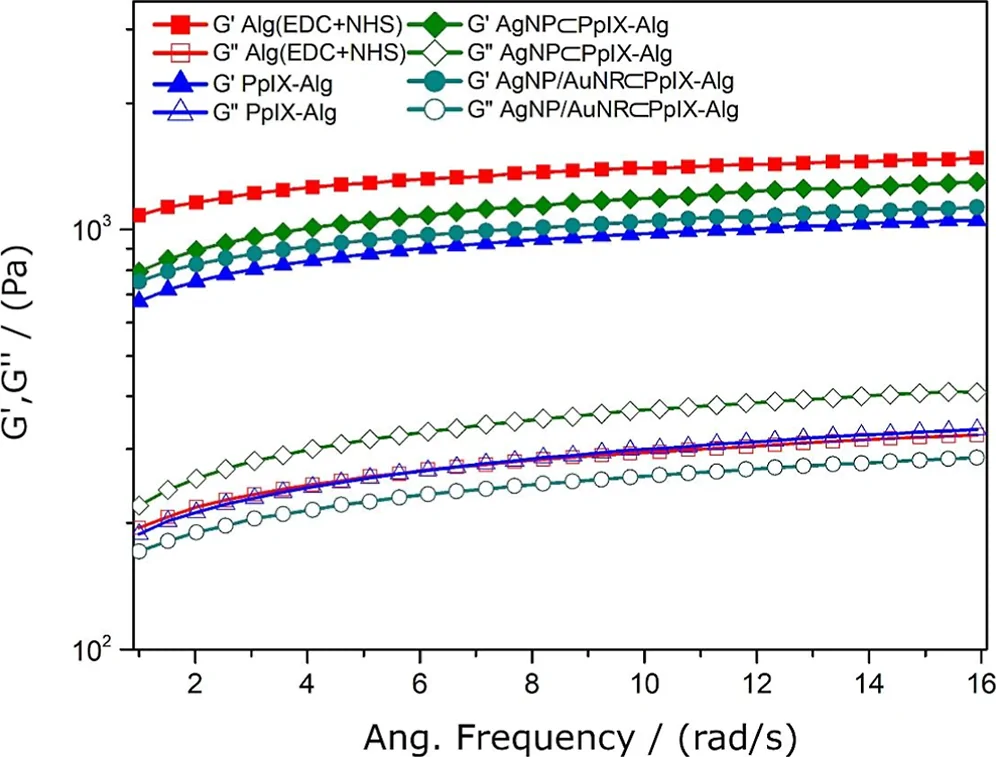
Frequency sweep experiments of PpIX-Alg, AgNP⊂PpIX-Alg, AgNP/AuNR⊂PpIX-Alg, and Alg (EDC + NHS).

After the conjugation reaction, the rheological properties became enhanced, where *G*′ became much greater than *G*″ in all conjugated materials. These were nearly independent of angular frequency over more than one and one-half decades of frequency. Moreover, the modulus values increased more than 1 order of magnitude with respect to the Alg gel material. This independence of the storage modulus from the frequency is a result of new cross-link formation between PpIX-Alg chains and with EDC and NHS, used to activate the carboxylic groups, leading to gel-like viscoelastic behavior.^75^ A further consequence was the increase of apparent viscosity and the maintenance of the non-Newtonian fluid behavior. The role of EDC and NHS as a cross-linking agent in the conjugated gels was verified in the Alg gel containing the mixture of both (EDC + NHS).

### 3.3 Thermal Analysis

Thermogravimetric analysis (TGA) was performed on all alginate-derived materials, as shown in Figure 8, after lyophilization. The thermal decomposition profile of alginate gel was significantly influenced by the mixture with EDC and NHS in the conjugated and hybrid gels, resulting in decreased thermal stability: the decomposition temperature of organic components decreased from approximately 210 °C (alginate gel) to around 170 °C (all other materials). Additionally, the presence of PpIX grafted onto the alginate chain altered the thermal decomposition above 270 °C. The mass loss observed at temperatures below 150 °C was attributed to the release of water molecules. All hybrid gels exhibited a similar decomposition profile to that of PpIX-Alg, indicating that the metal nanoparticles did not impact the thermal stability.

**Figure 8:**
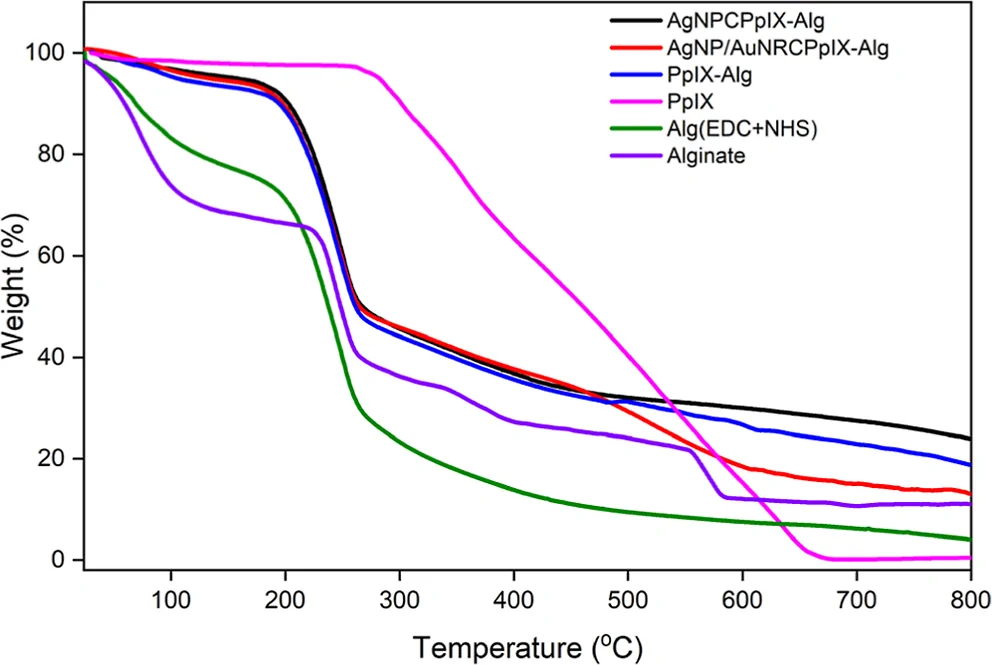
TGA of the gels AgNP⊂PpIX-Alg, AgNP/AuNR⊂PpIX-Alg, PpIX-Alg, PpIX, Alg­(EDC + NHS), and alginate.

### 3.4 Morphology

The scanning electron microscopy (SEM) images of PpIX-Alg (Figure 9a) and AgNP⊂PpIX-Alg (Figure 9b) reveal microscopic pores in the dried gels that are filled with water in the gel state. Importantly, the presence of MNPs and the conjugation with PpIX did not have an impact on the porosity of the dried gels. The high-resolution SEM image of AgNP⊂PpIX-Alg (Figure 9c) displays evenly distributed black points on the material’s surface. In the corresponding backscattered mode (BSEM) image (Figure 9d), these points appear white. In this acquisition mode, different materials are visualized with distinct contrasts.

**Figure 9:**
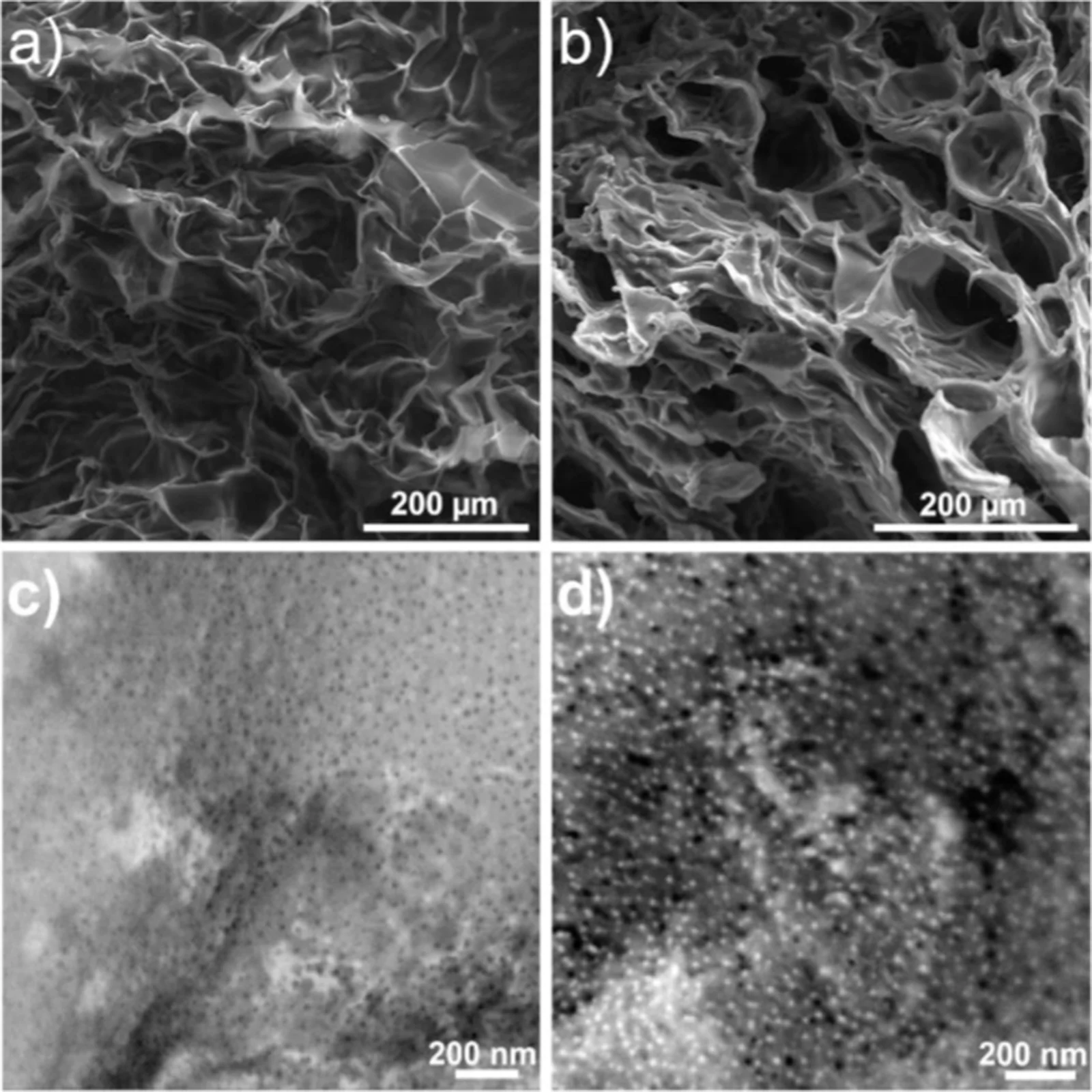
SEM images of a surface view of PpIX-Alg (a) and AgNP⊂PpIX-Alg (b–d).

Therefore, we can identify these points as AgNPs. The transmission electron microscopy (TEM) images of AgNPs and AgNP⊂PpIX-Alg (Figure 10) exhibit spherical nanoparticles with an average size distribution of 49 ± 12 nm (diagram in Figure 10a). Furthermore, we can conclude that the Alg gel matrix did not affect the size and structure of AgNPs.

**Figure 10:**
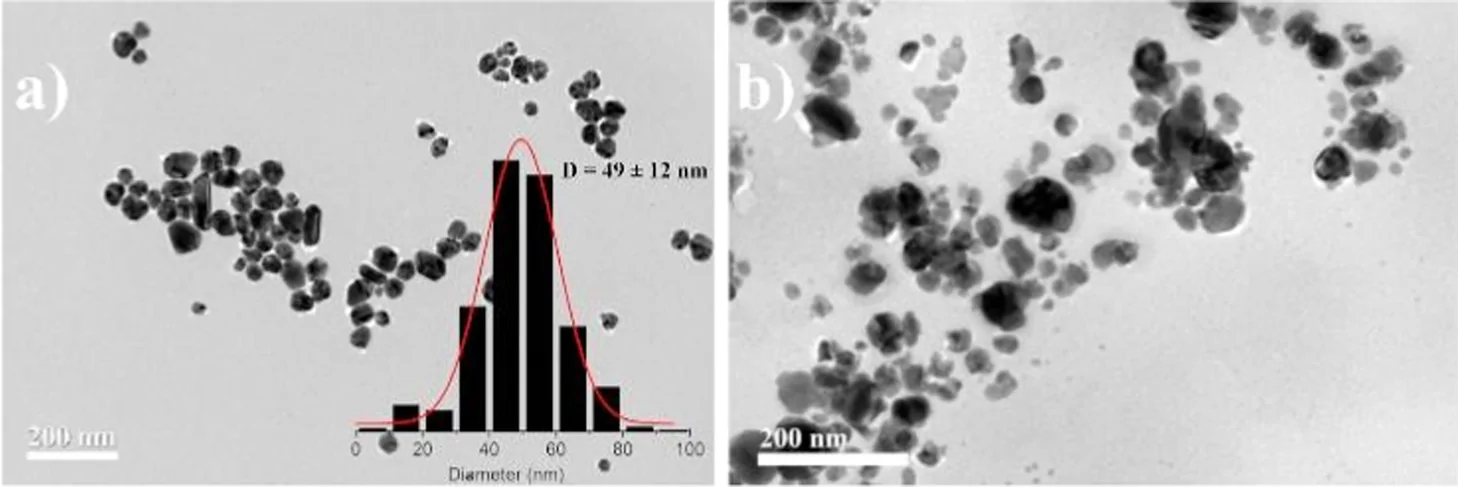
TEM image of the (a) AgNP suspension and (b) AgNP⊂PpIX-Alg hybrid gel. The insert in (a) is the size distribution diagram of AgNPs.

Figure 11 shows TEM images of AuNRs (a) and AgNP/AuNR⊂PpIX-Alg (b). The synthesized AuNRs display particles with an aspect ratio of 4.4 ± 0.1 (insert in Figure 11a). On the other hand, the TEM image of the AgNP/AuNR⊂PpIX-Alg hybrid (Figure 11b) displays nanospheres (AgNPs) and nanorods (AuNRs) similar to those found in Figures 10a and 11a, respectively. Also, the system exhibits a homogeneous and random dispersion of both spherical nanoparticles and nanorods throughout the matrix, without evidence of phase segregation or preferential clustering of a specific component. The observed distributions are consistent with those inferred from the SEM analysis of the AgNP⊂PpIX-Alg (Figure 9d) system and indicate that the incorporation of Au nanorods does not induce morphological changes to the AgNPs.

**Figure 11:**
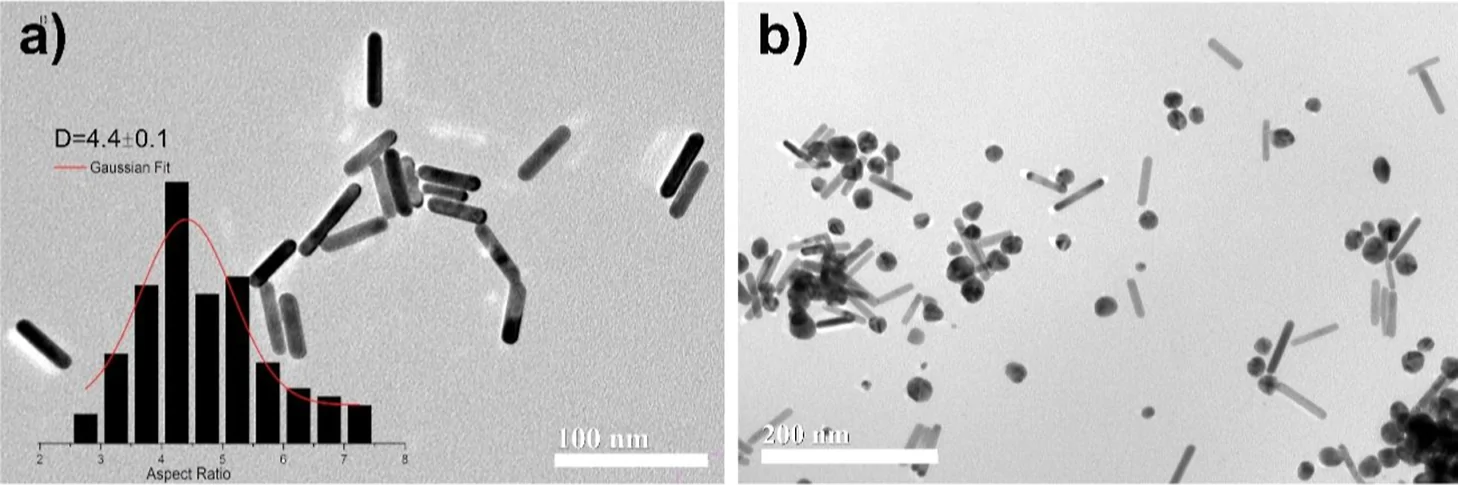
TEM image for the (a) AuNR suspension and (b) AgNP/AuNR⊂PpIX-Alg hybrid gel. The insert in (a) is the size distribution of AuNRs.

### 3.5 UV–Vis–NIR Spectroscopy

The absorbance spectra of MNP in Figure 12 exhibit one band (414 nm; black dotted line) and two bands (518 and 870 nm; black line) for AgNPs^76^ and AuNRs,^77^ respectively, related to Surface Plasmon Resonance (SPR) absorption. The SPR bands of AuNRs are assigned to the transverse (518 nm) and longitudinal (870 nm) surface plasmon resonance.^77^ The absorption spectrum of PpIX showed Soret (250–500 nm) and Q (500–750 nm) bands characteristic of porphyrin classes.^48,78–80^ Noticeable in the absorption spectra of the hybrids were absorption bands related to PpIX moieties overlapping with the SPR bands of the corresponding MNP, with, however, small deviations of the absorption maximum. This indicates that the gel environment does not affect the absorption related to SPR absorption of MNP. The slight shifts observed for the Soret and Q bands may be associated with changes in the local dielectric environment surrounding the PpIX molecules after incorporation into the alginate-based hybrid matrix. Additionally, intermolecular π–π interactions between neighboring porphyrin rings may contribute to slight spectral shifts and band broadening.

**Figure 12:**
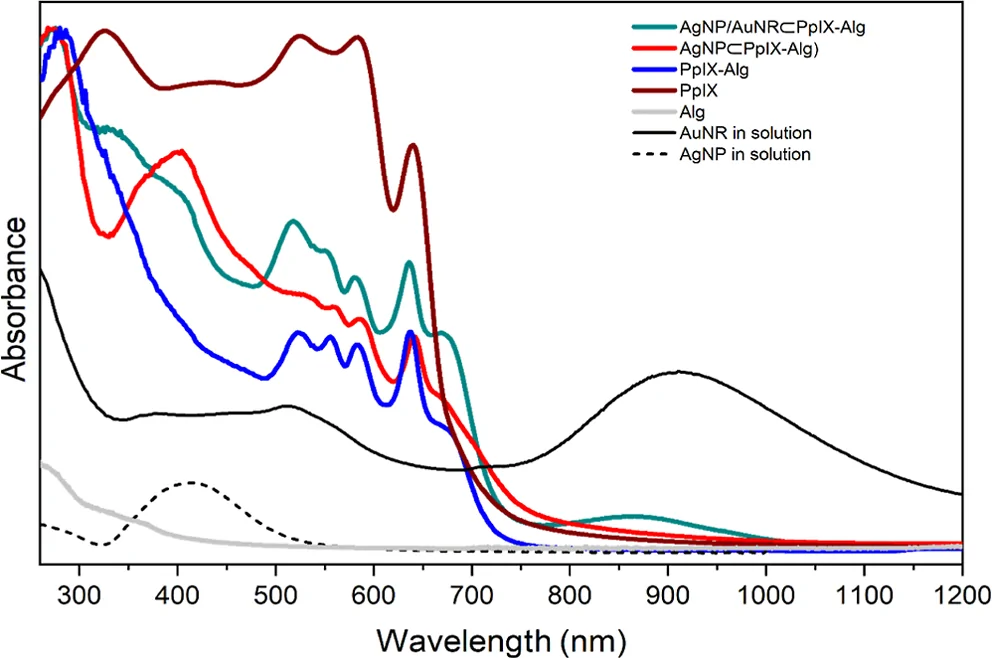
UV–vis–NIR spectra of AgNP/AuNR⊂PpIX-Alg, AgNP⊂PpIX-Alg, PpIX-Alg, PpIX, AuNRs in solution, AgNPs in solution, and alginate (Alg).

### 3.6 Singlet Oxygen Generation

To estimate the singlet oxygen quantum yield (SOQY), we used the chemical method (Figure 13), where DPBF was used as a singlet oxygen sensor by monitoring oxidization though absorbance at 414 nm. This was possible because DPBF reacts irreversibly with ^1^O_2_ and decreases the DPBF absorption at this wavelength.^50^ The numerical value of SOQY (Table 1) was estimated by means of eq 3

3 $$\Phi_{s}=\frac{K_{s}}{K_{R}}x\frac{{OD}_{R}}{{OD}_{s}}\Phi_{R}$$

where **Φ** is the SOQY of the sample (Φ_s_) and reference (Φ_R_), *k* is the slope of the curve plot of −ln­[DPBF]/[DPBF]_0_ vs *t* (irradiation time) for the sample (*K*_s_) and reference (*K*_R_), to each compound obtained after fitting with a linear function, OD is the optical density of the samples and reference at an irradiation wavelength of 660 nm (excitation source) as presented in Table 1. As a reference for a singlet oxygen generation sensitizer, we used an ethanolic solution of methylene blue, MB (Φ_R_ = 49%).^81–83^ All gel materials displayed a similar reduction rate of the absorption intensity of DPBF as a time function (Figures 13 and S7). Among these, AgNP⊂PpIX-Alg displayed a higher rate, indicating that this hybrid presented the highest singlet oxygen quantum yield (Table 1).

**Figure 13:**
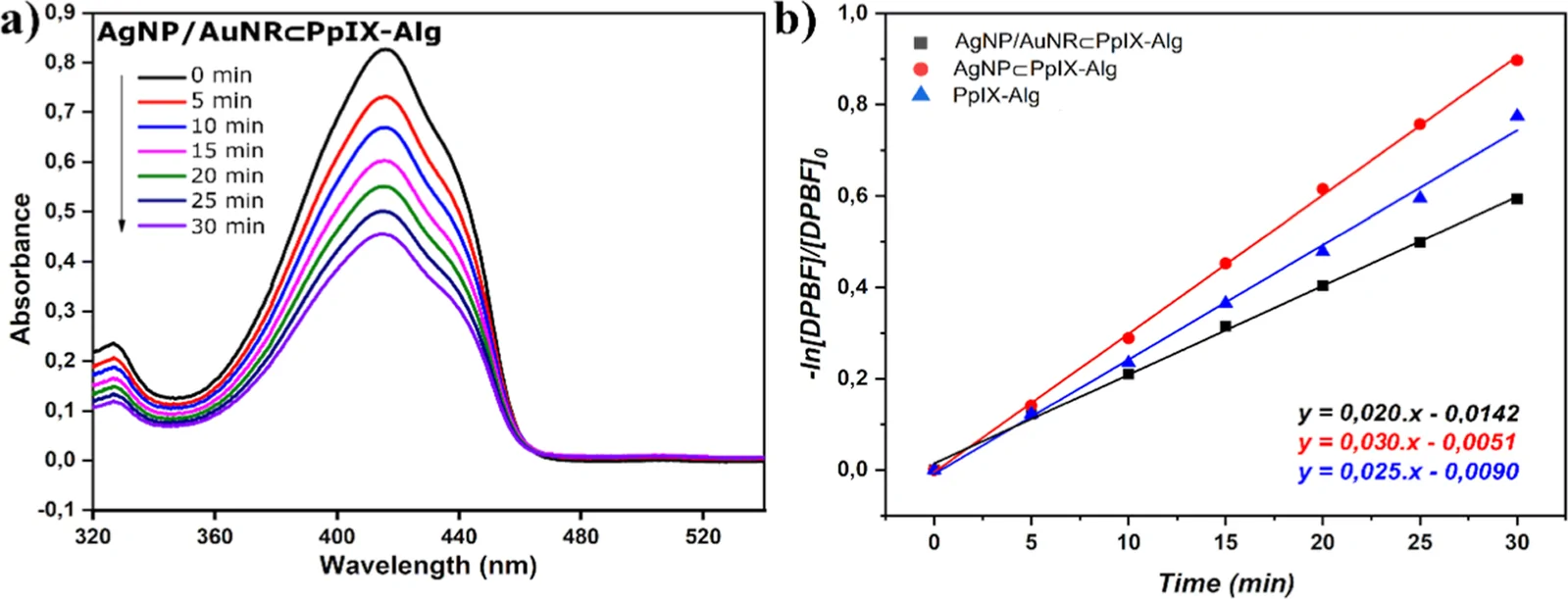
(a) Absorption spectra of the photodecomposition of the DPBF assay by singlet oxygen produced from the AgNP/AuNR⊂PpIX-Alg gel under irradiation with a 660 nm CW laser. (b) Variation of absorbance at 414 nm of DPBF vs irradiation time of AgNP/AuNR⊂PpIX-Alg during the photodecomposition assay.

**Table 1:** Singlet Oxygen Quantum Yield, Optical Density, and Slope of the Linear Regression of the Plot −ln­[DPBF]/[DPBF]_0_ vs Irradiation Time *t* of the Synthesized Materials and MB

photosensitizer	OD	*K*	SOQY (%)
PpIX-Alg	0.21	0.025	12.9
AgNP⊂PpIX-Alg	0.12	0.030	26.4
AgNP/AuNR⊂PpIX-Alg	0.12	0.020	17.5
MB	1.04	0.477	49

According to the SOQY data displayed in Table 1, the higher value displayed by AgNP⊂PpIX-Alg can be assigned to the action of the surface plasmon of the AgNP to improve the SOQY. On the other hand, AgNP/AuNR⊂PpIX-Alg has smaller SOQY values than AgNP⊂PpIX-Alg. This reduction is assigned to the absorption of AuNRs in the region of the excitation wavelength. In all systems, the PpIX moieties successfully acted as a photosensitizer, with the SOQY of AuNR⊂PpIX-Alg to a greater degree than other reported porphyrin-grafted nanomaterials.^84^

The difficult quantification of loaded photosensitizer PpIX in the hybrids by analyses such as TGA or ^1^H NMR represents a limitation of this study.

### 3.7 Photothermal Conversion

The performance of the photothermal transduction of the AgNP/AuNR⊂PpIX-Alg hybrid was evaluated by irradiation of the gel with a 785 nm CW laser. According to Figure 14, after the laser was turned on, the temperature rapidly increased for 300 s of irradiation, where it achieved equilibrium, suggesting that the material efficiently converted 785 nm light into thermal energy. This suggests that the material has great potential for cancer hyperthermia therapy.^35,85^ The photothermal transducer in this hybrid is the gold nanorods. As is well known, AuNRs exhibit strong absorption in the near-infrared (NIR) region and convert it into heat efficiently. For the assessment of the photothermal conversion efficiency of the AgNP/AuNR⊂PpIX-Alg hydrogel, it was exposed to a 785 nm NIR laser at an excitation power ranging from 0.5 to 1 W. Figure 14b shows the maximum variation of the equilibrium temperature (Δ*T*) of AgNP/AuNR⊂PpIX-Alg after irradiation for 5 min. The temperature rises from 27 to 45.5 °C as the power was turned from 0.5 to 1 W, respectively. These results indicate that no aggregation of AuNRs occurred with irradiation.^86^ On the other hand, with the PpIX-Alg and AgNP⊂PpIX-Alg systems under the same conditions as AgNP/AuNR⊂PpIX-Alg (1 W; 5 min), there were no significant temperature changes.

**Figure 14:**
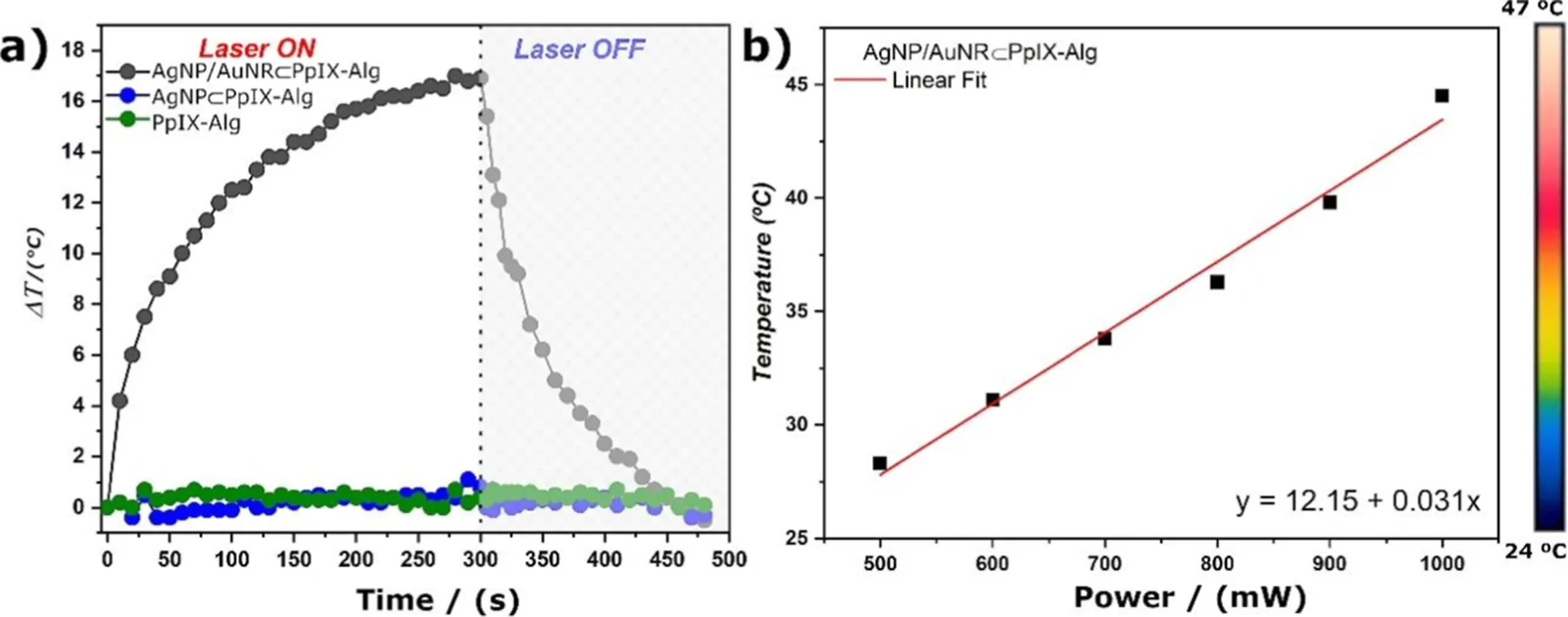
(a) Photothermal conversion performance of gels with irradiation with a 785 CW laser for 5 min. (b) Maximum temperature variation as a function of irradiation power, 500 to 1000 mW, with a CW 785 nm laser.

The photothermal conversion efficiency (η) was determined using eq 1, after irradiation with a 785 CW laser (1 W) for 5 min. From the plot of the cooling curve (180s) vs the negative natural logarithm of the driving force temperature (θ), a slope of 62.75s was obtained (Figure S8). The photothermal conversion efficiency (η) was estimated at 54.3%, indicating that the AgNP/AuNR⊂PpIX-Alg gel has a high potential as a photothermal agent in PTT.

### 3.8 In Vitro Assay of Photodynamic (PDT), Photothermal (PTT), and Chemo-Photothermal Therapy

To verify the safety of the photodynamic and photothermal effects, the cells were irradiated with 660 CWL (30 mW) and 785 CWL (800 to 1000 mW) (Figure 15).

**Figure 15:**
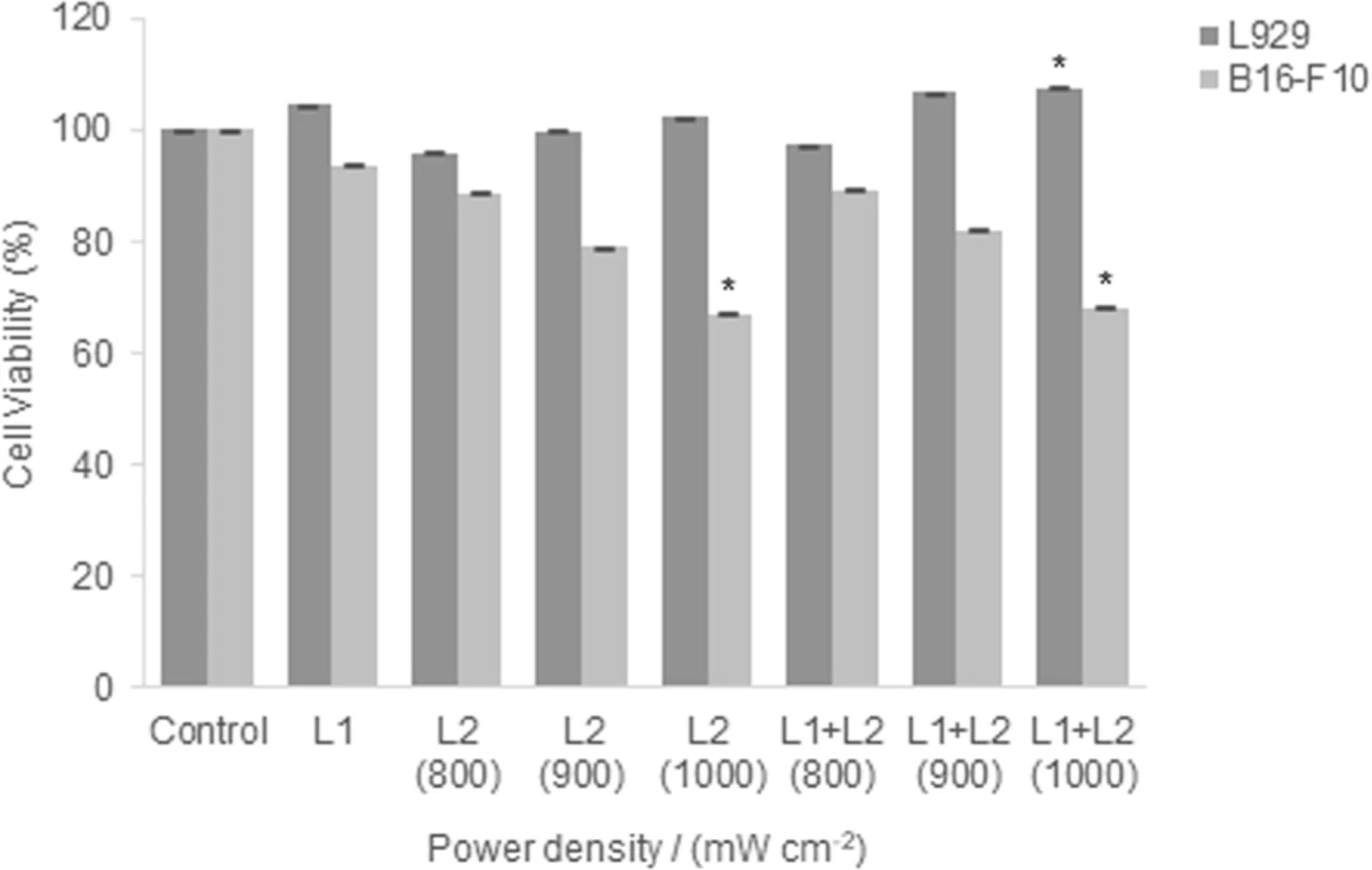
Phototoxicity of 660 CWL (L1) and 785 CWL (L2) on the cell viability of L929 and B16–F10 cells. The values are shown as mean ± SD; *p < 0.05. SD values ranged from 0.05 (L1) to 0.02 (L1 + L2) for L929 cells and from 0.04 (L1) to 0.08 (L1 + L2) for B16–F10 cells.

We observed that 660 CWL did not significantly reduce cell viability in the L929 and B16–F10 lines, showing viabilities of 104.60 and 93.77%, respectively (Figure 15). With respect to 785 CWL, tested at different power excitations, there was no significant effect on viability reduction in the L929 fibroblast cell line in the entire power range (varying from 95.84 to 102.27%). However, for the B16–F10 melanoma line, there was a significant reduction in cell viability at 1000 mW (67.02%).^52^ When both 660 CWL and 785 CWL were applied simultaneously, the response pattern was similar to that observed for 785 CWL, with a 68% reduction in viability at 1000 mW for B16–F10 melanoma cells, but with an increase of viability (107.63%) for L929 cells.

Results of cytotoxicity assays of PpIX and conjugated and hybrid gels without irradiation can be seen in Figure 16 for the L929 and B16–F10 cell lines.

**Figure 16:**
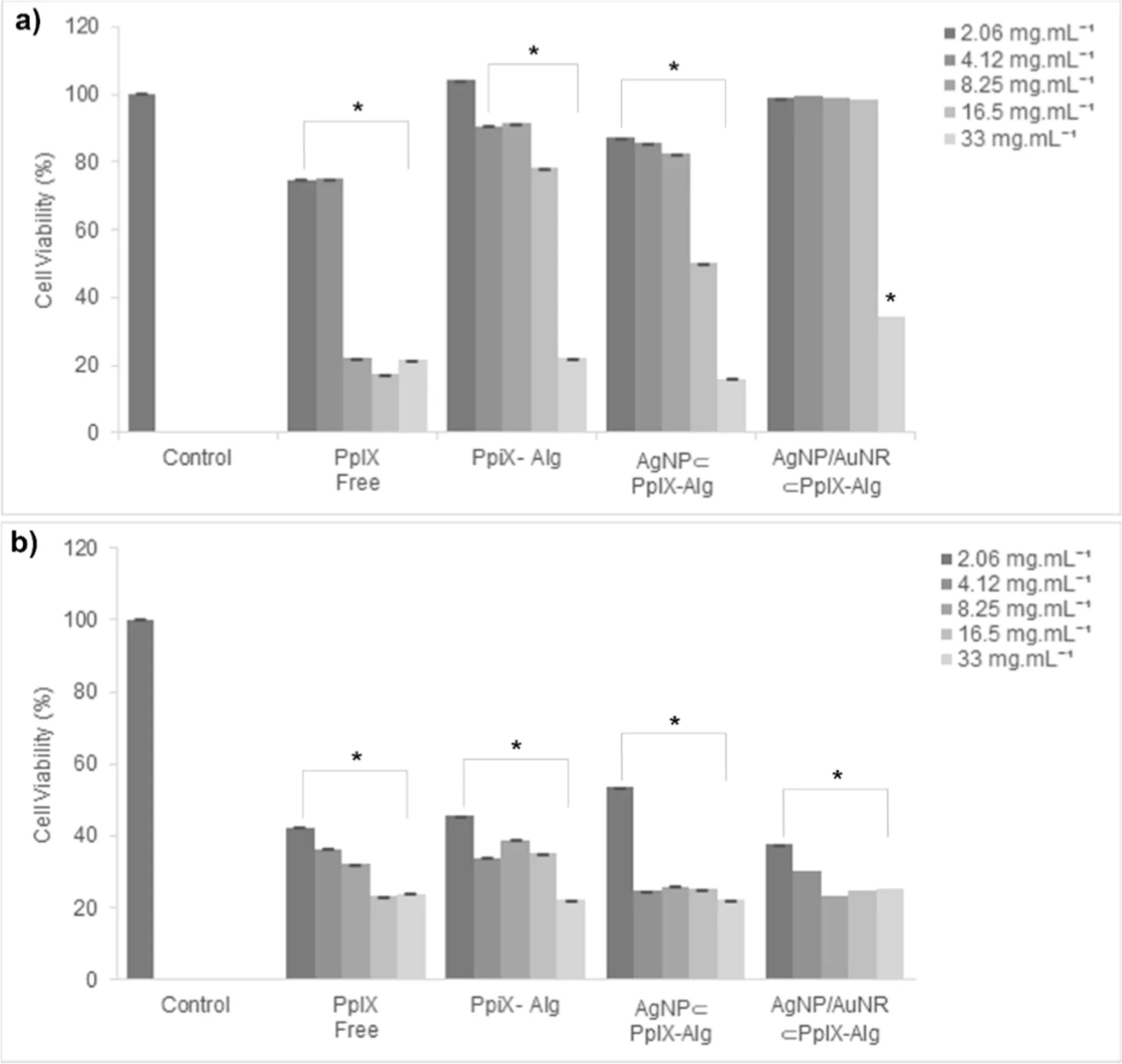
Cytotoxicity of PpIX, PpIX-Alg, AgNP⊂PpIX-Alg, and AgNP/AuNR⊂PpIX-Alg at different concentrations against L929 fibroblasts (a) and B16–F10 melanoma (b) cell lines after 48 h of exposure. Values are presented as mean ± SD; *p < 0.05. SD values ranged from 0.02 to 0.002 in L929 cells and from 0.08 to 0.01 in B16–F10 cells across the tested concentrations (2.0625–33 mg mL^–1^).

The PpIX solution significantly reduced cell viability in both L929 and B16–F10 cells, presenting cytotoxicity.^87^ However, when analyzing the conjugated and hybrid gels, PpIX-Alg, AgNP⊂PpIX-Alg, and AgNP/AuNR⊂PpIX-Alg, we found that the concentrations between 2.0 and 8.25 mg mL^–1^ showed cytotoxicity (viability less than 80%) only for the B16–F10 cell line, with viability ranging from 23.38 to 53.53%. The corresponding silver and gold concentrations in these solutions are listed in Table S3.

The concentrations 16.5 and 33 mg mL^–1^ were significantly cytotoxic for B16–F10 cells, with viability varying from 22.12 to 35.07%. At the concentration of 16.5 mg mL^–1^, PpIX-Alg and AgNP⊂PpIX-Alg reduced L929 cell viability to 78.29 and 50.16%, respectively. At 33 mg mL^–1^, the PpIX-Alg, AgNP⊂PpIX-Alg, and AgNP/AuNR⊂PpIX-Alg hybrid gels significantly reduced the L929 cell viability to 22.03, 15.81, and 34.31%, respectively. As observed in the experiments carried out, nanostructured photosensitizers managed to kill tumor cells without causing damage to healthy cells. They have selectivity for the melanoma cell line, which is interesting as it reduces adverse effects to nontumor cells.

According to Baffy et al., most cancer treatments produce very harmful side effects in the patient, especially the most debilitated, due to the cytotoxicity of drugs in nontumor cells.^85^ Currently, different types of carriers are being developed for the controlled delivery of a wide variety of drugs. Hydrogels have shown great potential to carry drugs through living organisms with low toxicity, as verified in the conjugated hybrid hydrogels developed in this work. In addition to bringing therapeutic benefits, they can also overcome cases of cancer drug resistance to multiple chemicals.^89^

To verify whether the conjugated hybrid gels had a satisfactory and synergistic effect of photodynamic and chemo-photothermal therapies, we selected AgNP⊂PpIX-Alg and AgNP/AuNR⊂PpIX-Alg at concentrations of 8.25 and 16.5 mg mL^–1^ for both cell lines (Figure 17).

**Figure 17:**
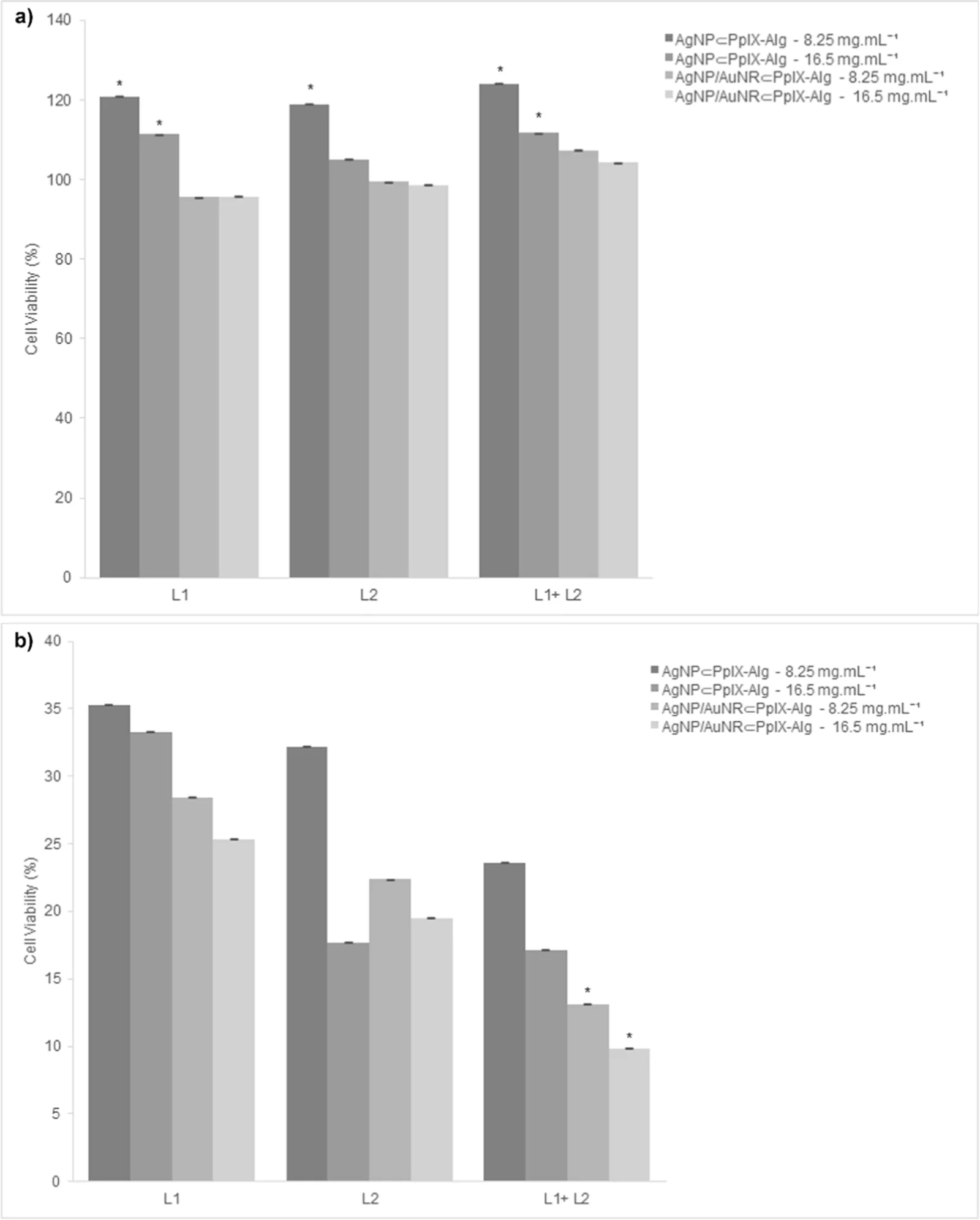
Photodynamic and chemo-photothermal therapy of the AgNP⊂PpIX-Alg and AgNP/AuNR⊂PpIX-Alg hybrid gels, irradiated with 660 CWL (L1, 30 mW), 785 CWL (L2, 1000 mW), and both simultaneously in (a) L929 and (b) B16–F10 tumor cells. Values are shown as mean ± SD; *p < 0.05. SD values ranged from 0.02 (L1) to 0.04 (L1 + L2) in L929 cells and from 0.004 (L1) to 0.001 (L1 + L2) in B16–F10 cells.

According to the results displayed in Figure 17, the use of 660 CWL resulted in a reduction in the viability of B16–F10 tumor cells ranging from 25 to 35% without reduction for L929 fibroblast cells. The selective reduction in B16–F10 cell viability under 660 nm irradiation may be associated with differences in photodynamic susceptibility between tumor cells and nontumoral fibroblasts.^71,91,101,102,103^ Since irradiation with the 660 nm laser alone did not significantly affect either cell line, the observed cytotoxic effect is more likely related to PpIX photoactivation and the subsequent generation of singlet oxygen and other reactive oxygen species (ROS). Upon light activation, photosensitizers can generate ROS through type I photochemical reactions (involving direct interactions between the photoexcited photosensitizer and cellular oxygen) and singlet oxygen through type II reactions (involving energy transfer from the photoexcited photosensitizer to molecular oxygen), thereby inducing oxidative stress and activating different cell death pathways.^105^ Cancer cells exhibit altered redox regulation, and although moderate ROS levels may support tumor progression, excessive ROS accumulation can exceed cellular tolerance, resulting in oxidative damage to biomolecules and ultimately leading to cell death.^106^ Therefore, the additional oxidative stress resulting from PpIX photoactivation may have contributed to the greater reduction in B16–F10 cell viability compared with L929 fibroblasts. This interpretation is consistent with the enhanced singlet oxygen generation observed for the AgNP-containing hybrid systems under 660 nm of irradiation. Future studies investigating intracellular ROS generation and cellular antioxidant responses may provide further insight into the molecular mechanisms underlying this differential cellular response.

Both irradiations, isolated or simultaneous, eliminated the cytotoxicity observed for L929 cells at 16.5 mg mL^–1^ AgNP⊂PpIX-Alg without irradiation (50.13%), presenting viability above 100% in the three photodynamic and chemo-photothermal therapies. Thus, our biomaterial proves to be an attractive nanotechnology platform, with the potential to overcome the nonspecific targeting, systemic toxicity, and development of resistance in cancer cells presented by other treatment proposals,^90^ demonstrating its potential solution by offering greater precision and effectiveness in the identification and destruction of cancer cells. This leads us to an effective immunotherapy.

We suggest that triggered hyperthermia is occurring inside out, a highly localized heating mechanism that rapidly destabilizes the plasma membrane. This mechanism mirrors the membrane disruption activity observed in photothermal hyperthermia with gold nanoparticles and may help to overcome chemoresistance by increasing drug access to the intracellular space, as demonstrated by Zamora-Pérez et al.^88^

The reduction in tumor cell viability caused by the hybrid gels AgNP⊂PpIX-Alg and AgNP/AuNR⊂PpIX-Alg irradiated with 660 CWL only occurred due to the photodynamic effect caused by increased singlet oxygen generation. It can be suggested that the localized surface plasmon resonance of the metal nanoparticle enhances the SOQY.^92^ In all samples irradiated with 785 CWL, the viability was more reduced, especially for the AgNP/AuNR⊂PpIX-Alg gel, whose viability was 19.51% at a concentration of 16.5 mg mL^–1^. This result was attributed to the heat generation due to the high photothermal conversion efficiency of AuNRs in the AgNP/AuNR⊂PpIX-Alg gel. AuNRs generate heat only when irradiated with 785 CWL, suggesting that photothermal therapy boosts the cytotoxicity of the material.^93^ After irradiation with both lasers (660 CWL and 785 CWL) simultaneously, we found that the AgNP/AuNR⊂PpIX-Alg gel at the tested concentrations (8.25 and 16.5 mg mL^–1^) significantly reduced tumor cell viability by 13.13 and 9.83%, respectively. These results confirm the effectiveness of the use of photodynamic and chemo-photothermal therapy of this conjugated hybrid gel, which suggests its use as a platform with high potential, therefore generating new alternatives for the treatment of skin cancer.^94^ When analyzing the combined photodynamic and chemo-photothermal therapy using both lasers simultaneously (660 CWL + 785 CWL), we observed a very promising result for the AgNP⊂PpIX-Alg and AgNP/AuNR⊂PpIX-Alg gels, as they had a synergistic effect in both therapies and resulted in a significant reduction of tumor cells. This synergism can be attributed to both singlet oxygen generation in the presence of NIR 660 CWL due to the presence of the conjugated PpIX photosensitizer in the gels,^36^ which caused acceptable photocytotoxicity, as well as the photothermal effect of AuNRs in the AgNP/AuNR⊂PpIX-Alg gel under NIR 785 CWL irradiation.^95–97^ Thus, photodynamic and photothermal therapies are indicated as having great potential for application against cancer.^98^ Many studies have shown that hyperthermia immediately after PDT increases the efficacy of both treatments. This is likely because of an inhibition of the cellular repair system caused by PDT or because hyperthermia also inhibits the repair response against PDT. There is also a synergistic effect since PDT induces mitochondrial damage, while hyperthermia induces damage to other organelles such as the nucleus and plasma membranes that are not oxidized by the singlet oxygen produced in the mitochondria.^99,100^

Recent studies have highlighted alginate-based biomaterials as promising platforms for photoactivated anticancer therapies due to their biocompatibility and ability to incorporate a wide range of therapeutic agents. In this context, Shi et al. developed an alginate-based hydrogel integrating poly­(*N*-isopropylacrylamide)-grafted dextran (dx)­(PNIPAM-*g*-dx) and loaded with doxorubicin (Dox), resulting in a multistimulus-responsive network for chemo-photothermal therapy, and observed a time-dependent reduction in the viability of HSC-3 cells (human oral squamous cell carcinoma), which decreased to below 30% after 10–15 min of NIR irradiation.^98^ Similarly, Gonçalves et al. reported a dual-cross-linked injectable alginate hydrogel loaded with doxorubicin and dopamine-reduced graphene oxide that reduced the viability of MCF-7 cells (human breast adenocarcinoma) to approximately 23%.^99^ This effect is attributed to the ability of photothermal heating to enhance doxorubicin release and increase tumor cell sensitivity to the drug. Likewise, Colak et al. developed 3D-printed alginate/CuS scaffolds that promoted synergistic photothermal, photodynamic, and chemodynamic effects.^100^ Following irradiation with an 808 nm laser, the viability of 4T1 cells (murine breast carcinoma) was reduced to approximately 10%, demonstrating that PDT and PTT processes can be simultaneously triggered by the Alg-CuS/BSA platform. Furthermore, Ni et al. developed a gelatin–alginate hydrogel containing Bi_2_Se_3_ nanostructures capable of combining photodynamic therapy, photothermal therapy, and ferroptosis induction.^104^ Treatment resulted in more than 80% tumor cell death in rat nasopharyngeal carcinoma cells (FAT) while preserving more than 95% viability of murine fibroblasts (L929) at a concentration of 100 μg mL^–1^. The authors attributed this effect to the enhancement of the antitumor activity of Bi_2_Se_3_ nanostructures under NIR irradiation. Collectively, these studies highlight the versatility of alginate as a functional matrix for integrating multiple phototherapeutic strategies. Although these platforms have demonstrated significant antitumor activity, some rely on the incorporation of chemotherapeutic agents, such as doxorubicin, or on the combination with additional therapeutic mechanisms, including chemodynamic therapy and ferroptosis, to maximize their therapeutic efficacy. In comparison, the AgNP/AuNR⊂PpIX-Alg hydrogel developed in the present study achieved a remarkably low cell viability when compared with recently reported photoactivated alginate-based platforms, reducing the viability of B16–F10 melanoma cells to only 9.83% following combined PDT/PTT treatment while maintaining low cytotoxicity toward L929 fibroblasts. Although direct quantitative comparisons should be interpreted with caution due to differences in cell lines, irradiation conditions, and therapeutic protocols, these studies collectively demonstrate the strong therapeutic performance of the present platform. Unlike several previously reported alginate-based photoresponsive platforms, whose efficacy relies on the incorporation of chemotherapeutic drugs such as doxorubicin or on additional therapeutic modalities, the AgNP/AuNR⊂PpIX-Alg hydrogel achieved a pronounced antitumor effect using only the synergistic combination of PDT and PTT. Therefore, the results obtained herein further support the translational potential of this platform for future applications in anticancer phototherapeutic strategies.

## 4 Conclusions

In summary, we report on a new multifunctional platform for the treatment of skin cancer. The porphyrin-conjugated hybrid gels were successfully obtained from the ethylenediamine bridge assisted by an EDC/NHS carboxyl activation agent, with EDC and NHS acting as the cross-linking agents to sodium alginate, as evidenced by rheological properties. The incorporation of AgNPs and AuNRs gives the porphyrin-conjugated gel noteworthy chemotherapeutic action and photothermal conversion performance (η = 54.3%), respectively. With the conjugation of porphyrin, the Alg gel achieved singlet oxygen generation (12.9% of PpIX-Alg, 26.4% of AgNP⊂PpIX-Alg, and 17.5% of AgNP/AuNR⊂PpIX-Alg) by 660 CWL irradiation. The in vitro assay showed that the AgNP/AuNR⊂PpIX-Alg gel can be used in combined PDT and PTT simultaneously. The results showed that cell viability of B16–F10 cells decreased significantly (∼9.83% remained) after the action of 660 CWL and 785 CWL lasers, without cytotoxicity for normal cells. Consequently, in vivo assays are warranted to ensure the safety of these biomaterials, specifically by assessing the synergistic effect preceding antitumor therapy.

## Supplementary Material



## Data Availability

Research data underlying this study are available in https://drive.google.com/drive/folders/1hIjiCOgHDWlCyfKneQqO_JJtm6Bye8LW?usp=drive_link.

## Notes

Volume of metal nanoparticle suspensions used to prepare the gel hybrids, structure of protoporphyrin IX, ^1^H NMR spectra of EDC, ^1^H NMR spectra of NHS, ^1^H NMR spectra of ethylene diamine, ^1^H NMR spectra of AgNP⊂PpIX-Alg, rheological analysis of hydrogels, monitoring the photodecomposition of DPBF, and fit cooling period (PDF)

## ¶

In memoriam.
