# Supplementary material for: Multifunctional Hybrid Gels Containing Silver Nanoparticles, Gold Nanorods, and Protoporphyrin IX for Synergistic Photothermal–Photodynamic Therapy
Source: ACS Omega. 2026 Aug 6;11(32):47711–29. doi: 10.1021/acsomega.6c02942 (PMC13491489; doi:10.1021/acsomega.6c02942)
Supplement: Supplementary file 1 [file ao6c02942_si_001.pdf]

Supporting Information

Multifunctional Hybrid Gels Containing Silver Nanoparticles, Gold Nanorods, and Protoporphyrin IX for Synergistic Photothermal–Photodynamic Therapy

Roberta C. S. Neves[a], Antônio L. de Oliveira[b], Silvany de S. Araújo[c, d], Palloma L. de Oliveira[c], Ana C. Brasileiro-Vidal[c], Iane B. V. Alves[e], José Y. R. Silva[e], Leonis L. Luz[e,g], Larissa T. Jesus [f], Ricardo O. Freire[, f], and Severino A. Junior[*a,e]*

[a] *Programa de pós-graduação em Ciências dos Materiais, Universidade Federal de Pernambuco UFPE, Recife, PE 50.740-560, Brazil.*
[b] *Departamento de Química da Universidade Estadual do Piauí, UESPI, Piripiri, PI 64.260-000, Brazil.*
[c] *Departamento de Genética, UFPE, Recife, PE 50.670-901, Brazil.*
[d] *In memoriam*
[e] *Departamento de Química Fundamental, UFPE, Recife, PE 50.740-560, Brazil.*
[f] *Pople Computational Chemistry Laboratory, Department of Chemistry, UFS, São Cristóvão - SE, 49107-230, Brazil.*
[g] *Núcleo Interdisciplinar de Ciências Exatas e da Natureza, CAA, Universidade Federal de Pernambuco, Nova Caruaru, Caruaru, PE, 55.014-900, Brazil*

*Corresponding author: rfreire@academico.ufs.br ORCID 0000-0002-3603-3184
severino.alvesjr@ufpe.br ORCID 0000-0002-8092-4224

Table of contents

Equipment descriptions…………………………...…………………….…………………………………..…...	2
**Table S1.** Volume of metal nanoparticle suspensions used to prepare the gels hybrids.................	3
**Figure S1.** Chemical Structure of Protoporphyrin IX Indicating hydrogens 3a, 8a, 3b and 8b.....	4
**Figure S2.** ^{1}H NMR spectra of EDC ……………………………………………………………...	4
**Figure S3.** ^{1}H NMR spectra of NHS ……………...……………………………………………..	5
**Figure S4.** ^{1}H NMR spectra of ethylene diamine.......................…………….…………........	5
**Figure S5.** ^{1}H NMR spectra of AgNPPpIX-Alg…………….…………………………….….....	6
**Figure S6.** Rheological analysis of hydrogels of: a) Alginate, (4% mass of alginate) measured by of storage modulus (G') and loss modulus (G") versus oscillation frequency (rads/s). b) Alginate, AgNP-Alg and AgNP/AuNR-Alg at (4% mass of alginate), measured by shear viscosity as a function of shear rate at plate temperature of 25°C……………………………….………..	6
**Figure S7.** Monitoring the photodecomposition of DPBF in the maximum absorption of 414 nm, band in the presence gels hybrids of a) AgNPPpIX-Alg and b) PpIX-Alg…………………..........	7
**Figure S8.** Fit cooling period (180 s) versus the negative natural logarithm of the driving force temperature(Θ)………………….……………………………………………………………….…....	7

Equipment descriptions

The morphological analyses of Alg and hybrid gels were evaluated with a scanning electron microscopy (SEM), TESCAN MIRA3 FEG at a voltage of 10 kV, and with a transmission electron microscope (TEM) thermos-scientific Joel JEM-2100 F instrument (200 kV). The ultraviolet-visible-near infrared (UV-vis-NIR) diffuse reflectance spectra were obtained on a spectrophotometer UV-2600-Shimadzu. The absorption spectra were obtained by the Kubelka-Munk approach [F(R)=(1-R)2/2R]. Thermogravimetric analysis was carried out in a DTG-60H from Shimadzu in a continuous flow of nitrogen and a heating rate of 10 °C/min. FTIR spectra were acquired on a NEXUS-870 spectrophotometer (Perkin Elmer model Spectrum 400), in a frequency range from 4000 to 400 cm^{-1}, through the FT-IR method with UATR accessory. ^{1}H-NMR spectra were measured in a VARIAN Unity Plus, operating at 300 MHZ. The samples were solubilized in D_2O (Alg gel material) or DMSO-d6 (all other materials) at room temperature. Rheological properties, at room temperature (25 °C), were performed on a stress-controlled rheometer DHR from TA Instruments, using software Rheometer MCR 301 - Anton Paar Physica. All measurements were made using parallel plate geometry (50 mm diameter) at a gap of 100 µm. Oscillatory dynamics measurements were performed in the frequency scan mode, where the applied stress (τ) was constant 0.4 or 5 Pa in the domain of the linear viscoelastic region and the oscillation frequency (ω) (0.1 to 100 Hz). The excitation sources used in the experiment of SOG and PTT used a DMCABC Photon laser III and a Laser Multimodo I0785MB0600MS respectively.

Silver and gold quantification

To estimate the Ag and Au concentrations, we have used spectrophotometric methods. Firstly, the concentrations of silver nanoparticles (AgNPs) and gold nanorods (AuNRs) were estimated from their localized surface plasmon resonance (LSPR) absorption spectra according to the Beer–Lambert law:

$$A = Q_{Ex} \cdot c_{NP} \cdot l$$

where A is the absorbance at the plasmon resonance maximum, Q_{Ex} is the particle molar extinction coefficient, c_{NP} is the nanoparticle concentration, and l is the optical path length (1 cm).

For AgNPs, the nanoparticle concentration was determined using the tabulated molar extinction coefficients reported by Paramelle et al. for citrate-stabilized spherical silver nanoparticles, which correlate the extinction coefficient with the LSPR wavelength and particle diameter. Although transmission electron microscopy (TEM) yielded an average particle diameter of 49 nm, the extinction coefficient was selected according to the measured plasmon resonance maximum (412 nm), which corresponds to an effective optical diameter of approximately 40 nm in the calibration reported by Paramelle et al.[1] The extinction coefficient used is 3.36×10^{10} L mol^{-1} cm^{-1}, and the absorbance at 412 nm is A = 0.5.

The total silver concentration was subsequently calculated from the nanoparticle concentration using the estimated number of silver atoms per nanoparticle, obtained from the particle volume assuming spherical geometry and the bulk density of metallic silver (ρ = 10.49 g cm^{-3}).

$$[Ag] = c_{NP} \cdot N_{Ag},$$

where $N_{Ag} = N_A\left(\frac{\rho \cdot V_{Esf}}{M}\right)$, and $V_{Esf} = \frac{4\pi r^3}{3}$

Thus,

$$[Ag] = c_{NP} \cdot N_A \left(\frac{4 \cdot \rho\pi r^3}{3 \cdot M_{Ag}}\right)$$

N_{Ag} is the number of silver atoms, V_{Esf} and r are the volume and radius of the nanoparticle, respectively, N_A is the Avogadro number, and M_{Ag} is the atomic weight of the silver. The concentration of silver in the AgNP suspension is presented in Table S2.

For AuNRs, the nanoparticle concentration was estimated using the relationship between the longitudinal plasmon extinction coefficient and nanorod aspect ratio established by Orendorff and Murphy.[2] Considering the measured aspect ratio (4.4) and the longitudinal plasmon resonance absorbance centered at 910 nm (A = 1.35), the corresponding molar extinction coefficient was obtained by linear extrapolation of the published calibration curve.

The total gold concentration was then calculated from the nanoparticle concentration using the average nanorod dimensions determined by TEM (49 × 11 nm), assuming a cylindrical body capped by two hemispherical ends and the bulk density of metallic gold.

$$[Au] = c_{RN} \cdot N_{NR},$$

where $N_{NR} = N_A\left(\frac{\rho \cdot V_{NR}}{N_{Au}}\right)$, and $V_{NR} = \pi r^2 l_c + \frac{4}{3}\pi r^3$

Thus,

$$[Au] = c_{NR} \cdot N_A \left(\frac{\rho}{N_{Au}}\right)\left(\pi r^2 l_c + \frac{4\pi r^3}{3}\right)$$

N_{NR} is the number of gold atoms, V_{NR} and r are the volume and radius (r = 11/2 = 5.5 nm) of the nanorod, respectively, N_A is the Avogadro number, l_c is the cylindrical length of the nanorod (lc = 49 - 11 = 38 nm), and

M_{Au} is the atomic weight of the gold. The concentration of gold in the AuNR suspension is presented in Table S2.

Table S1. Volume of metal nanoparticle suspensions used to prepare the gels hybrids.

Sample \ Particles	Volume	
	**AuNR**	**AgNP**
AgNP□PpIX-Alg	-	4 mL
AuNR/AgNP□PpIX-Alg	4 mL	4 mL

Table S2. Concentrations of Ag and Au in the hybrid gels.

Material	[Ag] (μg mL^{-1})	[Au] (μg mL^{-1})
AgNP	3.13	-
AuNR	-	11.00
AgNP□PpIX-Alg	2.50	-
AuNR/AgNP□PpIX-Alg	1.39	4.89

Table S3. Correlation of concentrations of the gel hybrids and Ag and Au content.

Material	[Gel] mg mL^{-1}	[Ag] (ng mL^{-1})	[Au] (ng mL^{-1})
AgNP□PpIX-Alg	2.06	4.12	-
	4.12	8.24	-
	8.20	16.4	-
	16.50	33.00	-
	33.00	66.00	-
AuNR/AgNP□PpIX-Alg	2.06	2.50	8.85
	4.12	5.03	17.7
	8.20	10.06	35.4
	16.50	20.13	70.8
	33.00	40.25	141.6

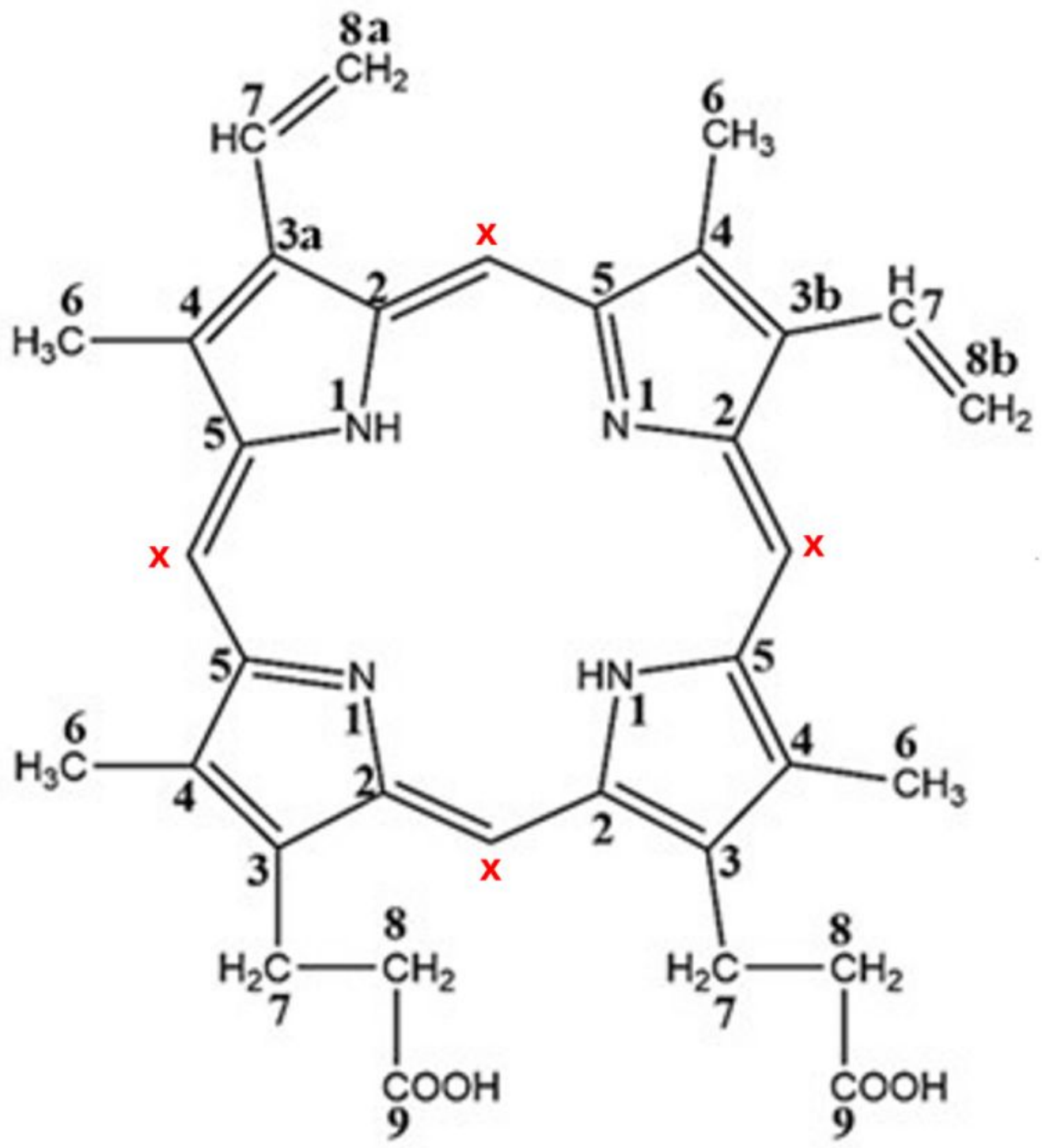

Figure S1. Chemical Structure of Protoporphyrin IX Indicating hydrogen positions linked to x, 6, 7, 8, 8a, 8b, 9 carbons.

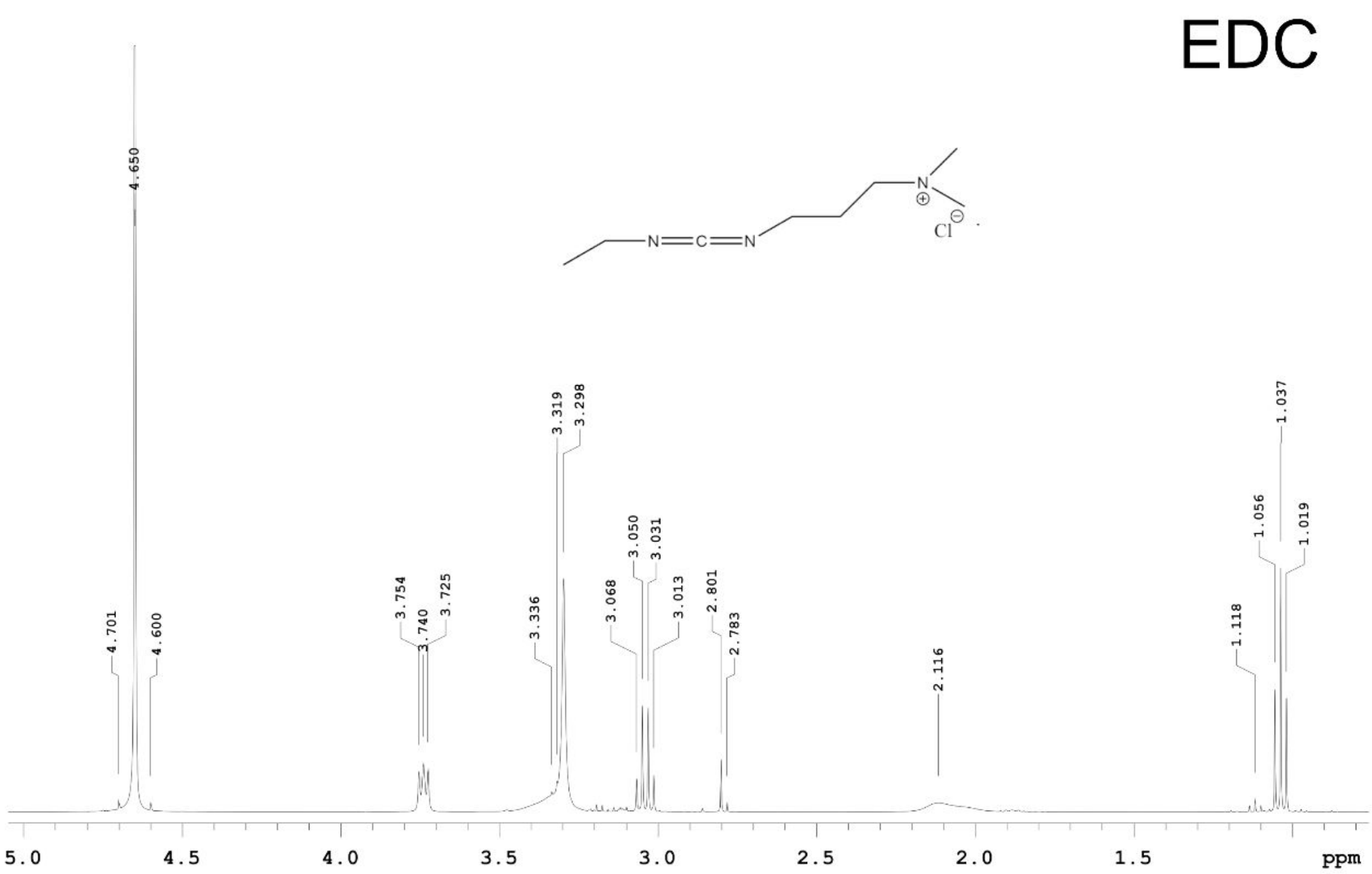

Figure S2. ^{1}H NMR spectra of EDC.

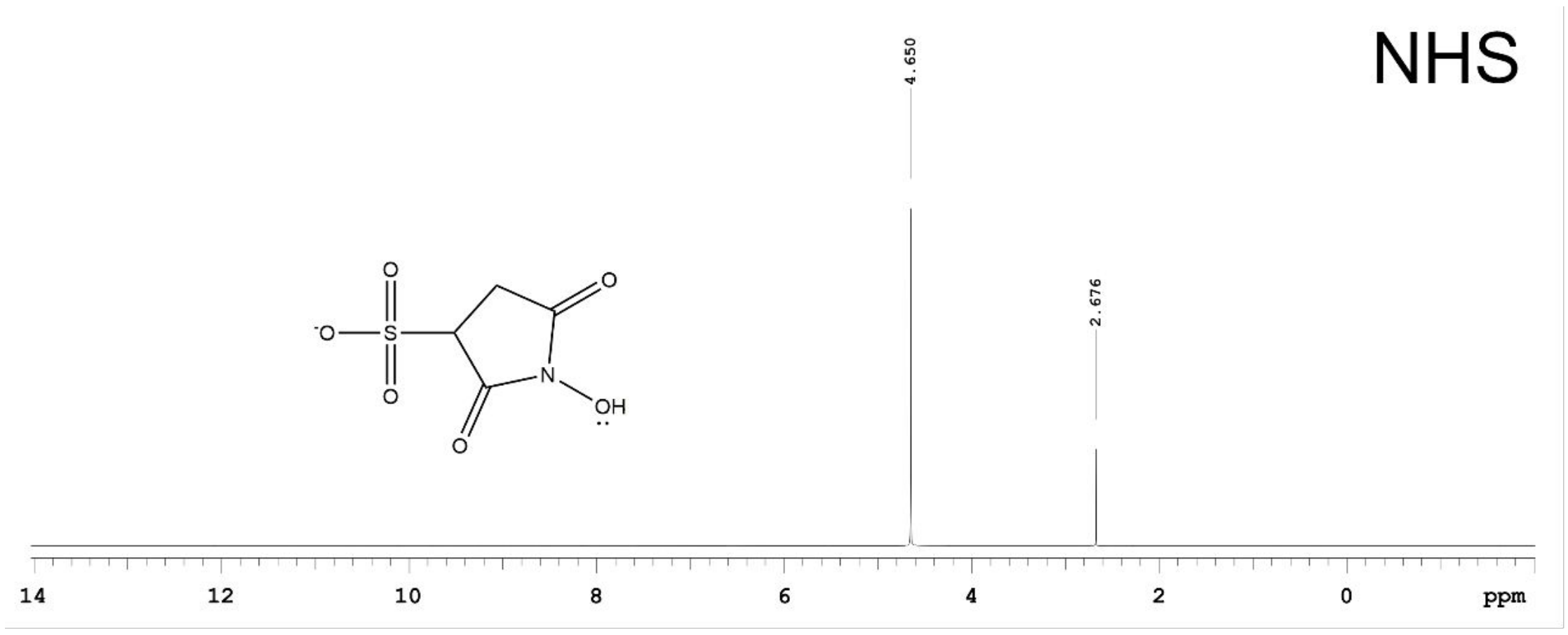

Figure S3. ^1H NMR spectra of NHS.

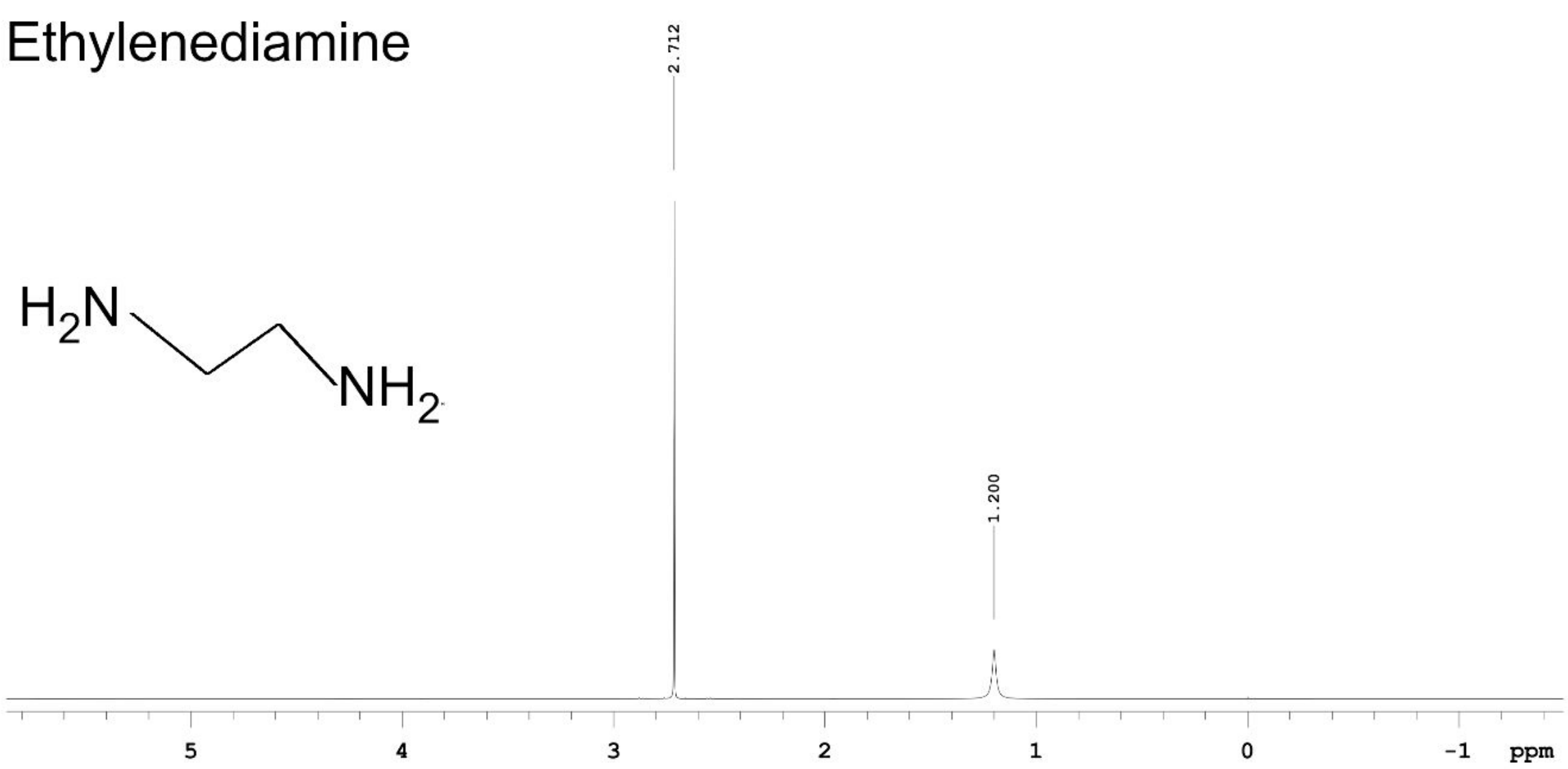

Figure S4. ^1H NMR spectra of ethylene diamine.

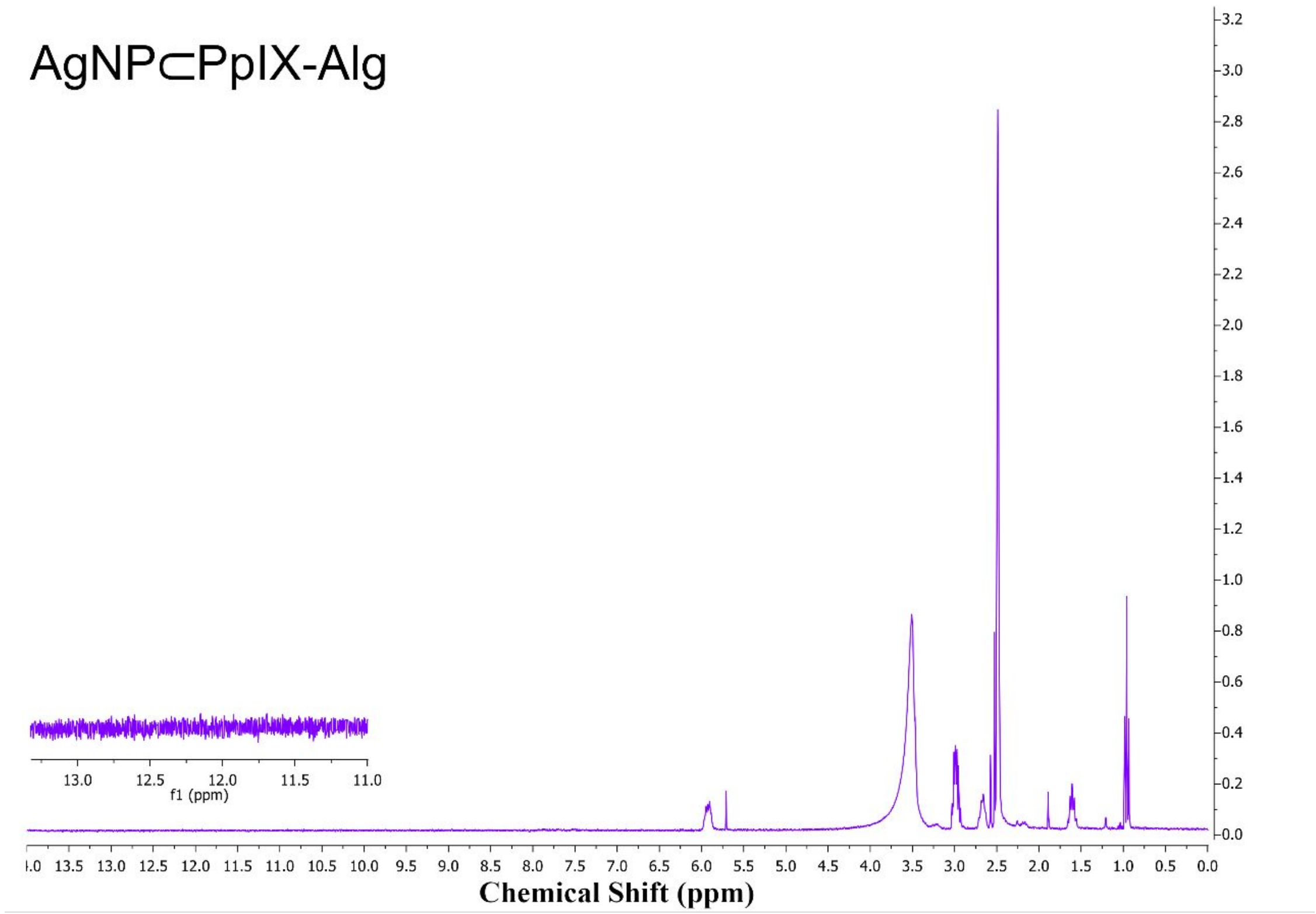

Figure S5. ^{1}H NMR spectra of AgNP□PpIX-Alg.

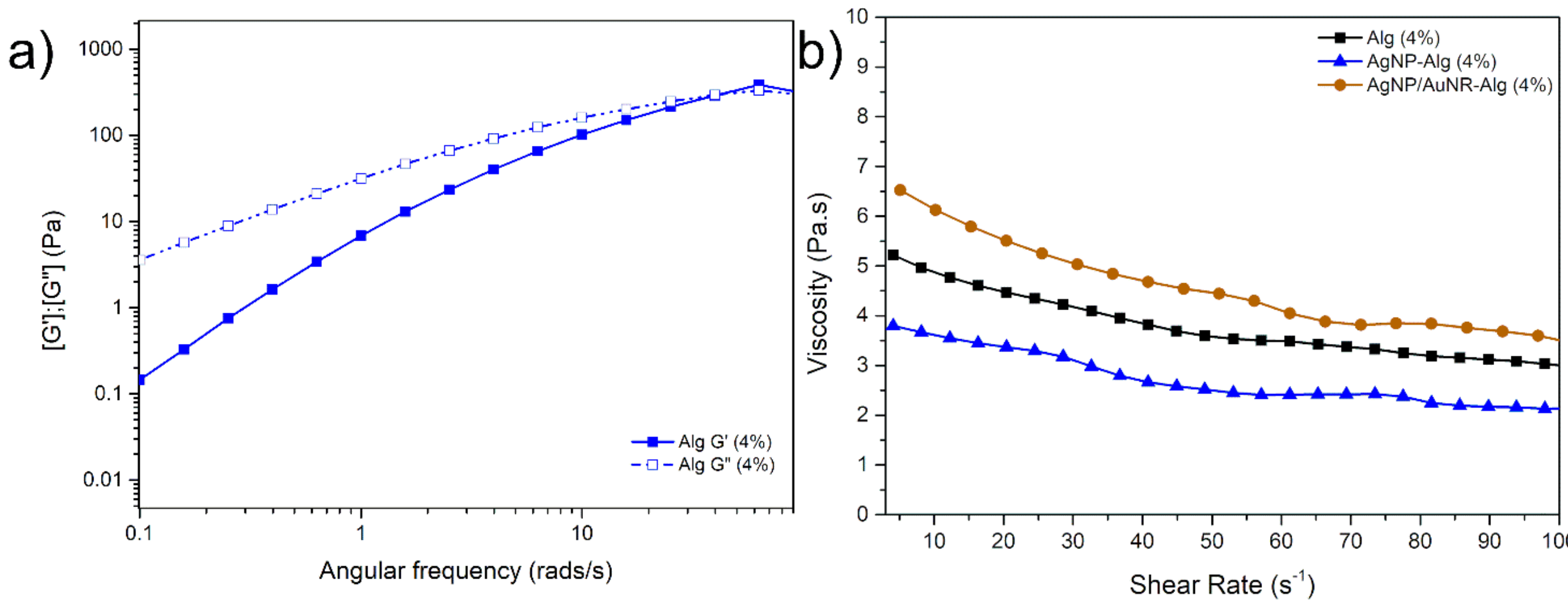

Figure S6. Rheological analysis of hydrogels of: a) Alginate, (4% mass of alginate) measured by of storage modulus (G') and loss modulus (G") versus oscillation frequency (rads/s). b) Alginate, AgNP-Alg and AgNP/AuNR-Alg at (4% mass of alginate), measured by shear viscosity as a function of shear rate at plate temperature of 25°C.

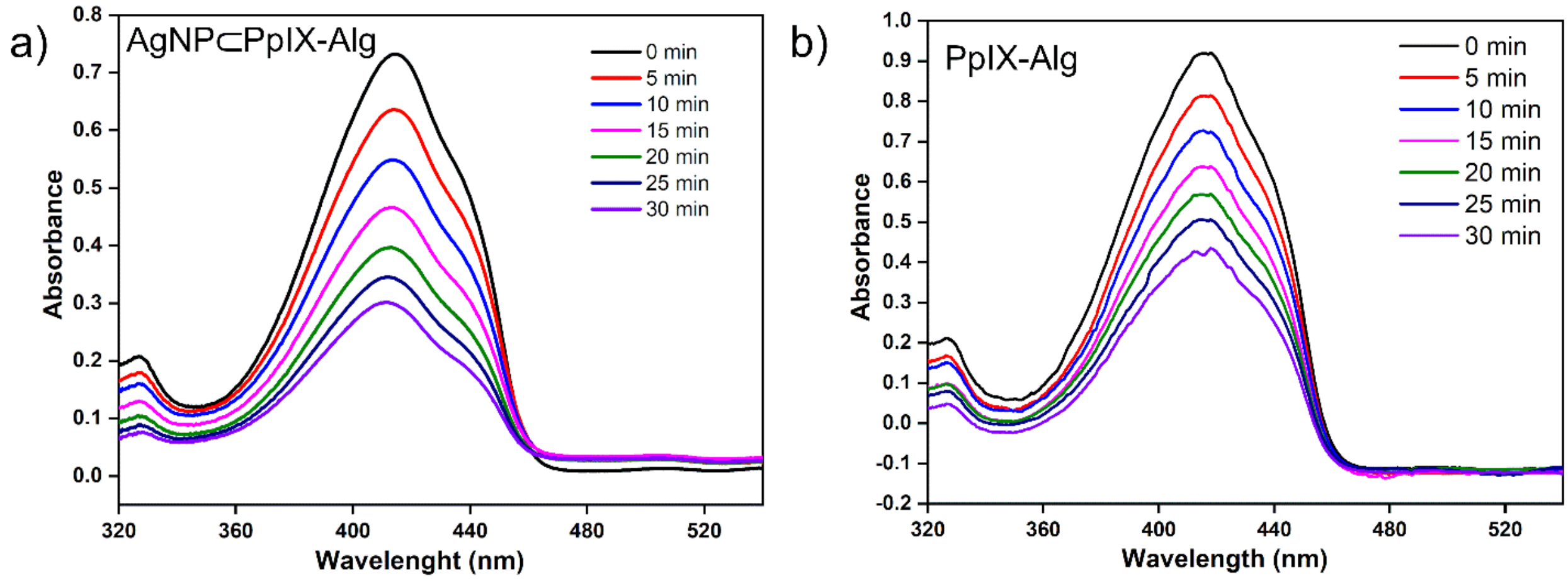

Figure S7. Monitoring the photodecomposition of DPBF in the maximum absorption of 414 nm, band in the presence gels hybrids of a) AgNP□PpIX-Alg and b) PpIX-Alg.

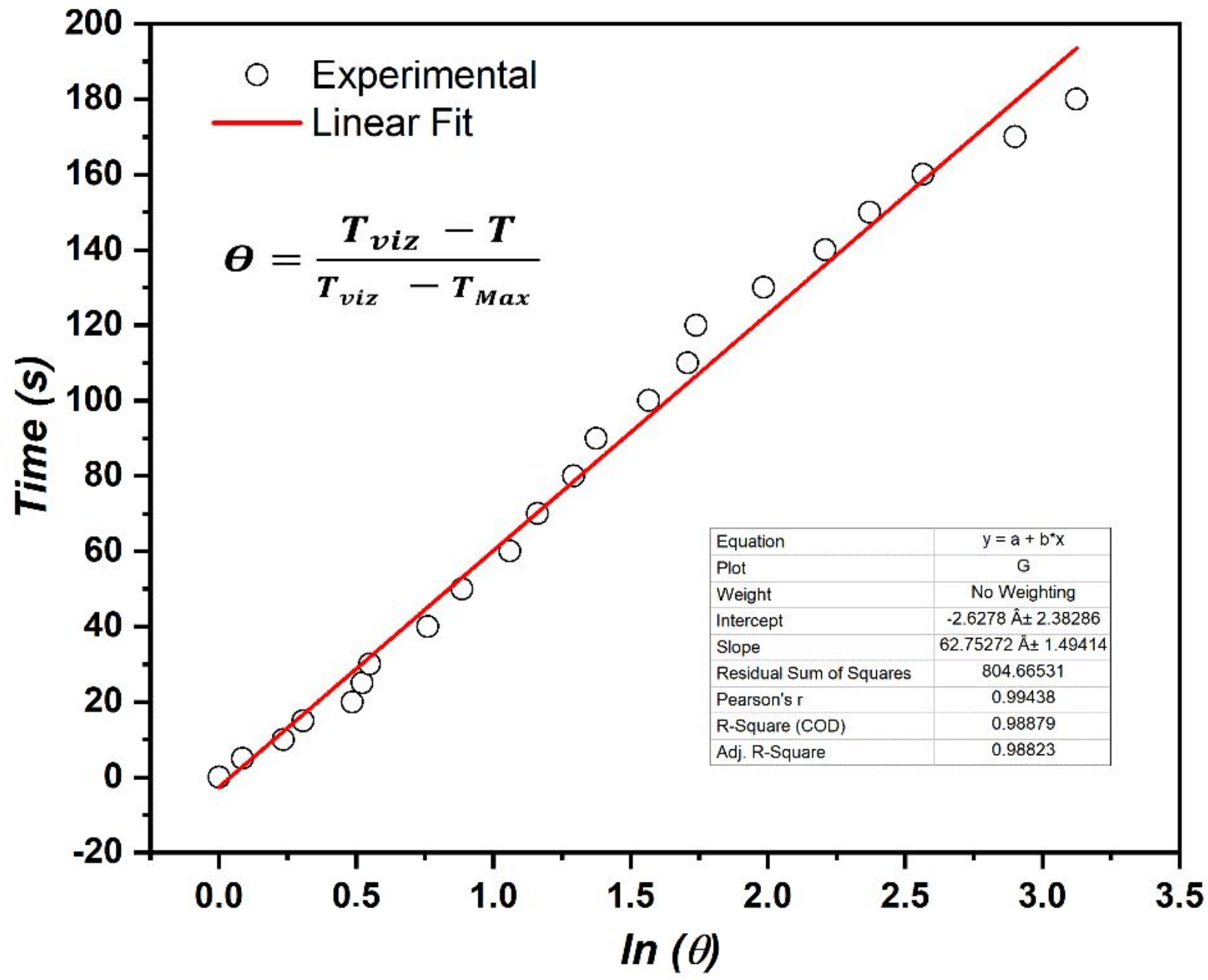

Figure S8. Fit cooling period (180 s) versus the negative natural logarithm of the driving force temperature (Θ).

References

(1) Paramelle, D.; Sadovoy, A.; Gorelik, S.; Free, P.; Hobley, J.; Fernig, D. G. A Rapid Method to Estimate the Concentration of Citrate Capped Silver Nanoparticles from UV-Visible Light Spectra. Analyst 2014, 139 (19), 4855–4861.

(2) Orendorff, C. J.; Murphy, C. J. Quantitation of Metal Content in the Silver-Assisted Growth of Gold Nanorods. J. Phys. Chem. B 2006, 110 (9), 3990–3994.
